# PegaPlus—Interactive Machine Learning by Human Observation for Efficient Clustering and Analysis of Structure–Activity Data

**DOI:** 10.1021/acs.jcim.6c00524

**Published:** 2026-06-23

**Authors:** Rainer Fährrolfes, Jochen Sieg, Florian Flachsenberg, Matthias Rarey

**Affiliations:** † 14915 University of Hamburg, ZBH—Center for Bioinformatics, Albert Einstein Ring 8-10, 22761 Hamburg, Germany

## Abstract

In the early stages of drug discovery, the identification of molecular series with lead-like properties is essential for structure–activity relationship studies. A common first step is to cluster data derived from high-throughput screening (HTS). This data then needs to be exhaustively refined by an expert to identify bioactive compound classes. Here, we present the novel, interactive, and visual approach named PegaPlus for the analysis of HTS data, facilitating the incorporation of expert knowledge with the strategy of learning by observation. PegaPlus uses a tailored variant of a stochastic proximity embedding algorithm to visualize the clustered data in a two-dimensional plane via a web interface. The user can iteratively modify the proposed compound clustering by dragging data points in the drawing plane. At each step, an online support vector machine learns and improves its clustering predictions by incorporating user modifications and the molecular features of the compounds. The visualization is also updated based on the user’s input and molecular similarity. PegaPlus was developed with the exploratory process in mind and allows the user to freely organize molecules on the drawing plane, or filter them by various molecular properties. Our evaluation shows that PegaPlus reduces the number of refinement steps needed by half in comparison to the purely manual approach on multiple data sets. These results demonstrate how an interactive machine learning approach can reliably support medicinal chemists and automate time-consuming, manual refinement tasks. The PegaPlus web server is openly available at pegaplus.zbh.uni-hamburg.de.

## Graphical abstract

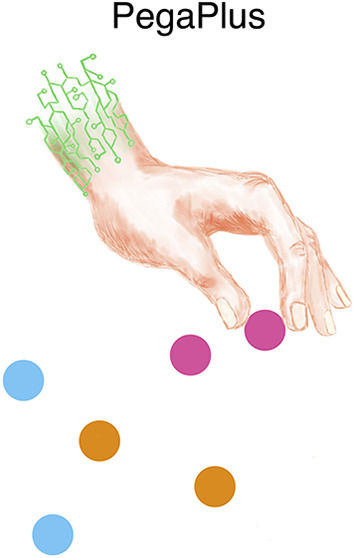

## Introduction

In the early phase of drug discovery, the accumulated knowledge and experience of medicinal chemists are invaluable for prioritizing drug candidates.^1^ Enabling experts to provide their feedback during computational processes is a promising direction, not only to build better machine learning models but also to make practitioners more effective and efficient in their daily tasks. Human-in-the-loop machine learning, like active learning and interactive machine learning, provides a set of methods and strategies to connect expert knowledge with computational models.^2^

Active learning (AL) has been applied in various processes in the early stages of drug discovery.^3–8^ The goal of AL is to achieve better performance with less training.^9^ This makes AL approaches interesting for areas where labeled data are scarce or the labeling process is expensive. A common approach in AL is training a machine learning model iteratively, querying a pool of unlabeled samples for annotation through an oracle, e.g., a human annotator or experimental testing in the wet lab. There are different sampling and querying strategies to select unlabeled samples for annotation.^2,9^ The hypothesis is that a good selection of samples for labeling reduces the number of labeled samples needed and, therefore, the overall cost of training.^9^

One of the first and most prominent applications of AL in drug discovery has been sequential (virtual) screening.^10–16^ The AL paradigm has similarities with the design-make-test-analyze cycle of the drug discovery process, in which the next iteration of compounds to be tested experimentally is selected based on the knowledge of all available compounds already evaluated.^5,6^ Over the years, AL has been applied to various problems in drug discovery. For example, extracting the intuition of medicinal chemists about which compounds are preferable,^17^ exploring the vast chemical space in make-on-demand libraries more efficiently,^18,19^ and the targeted improvement of property predictors during the exploration of a new chemical space with generative models.^20^

Developing systems that incorporate human feedback has further challenges in addition to the development of a proper algorithm. Usually, a reasonable design of the human-computer interaction is needed, including a graphical user interface (GUI) and an appropriate question design to prompt the required information from the user. For example, Choung et al.^17^ prepared a GUI to let the user choose which of two compounds they prefer. Metis^21^ is a GUI used by Nahal et al.^20^ where the user shares how strongly they agree with a predicted molecular property. Even with a user-friendly GUI in place, asking the right question with the necessary specificity or generality^20^ or with unambiguous examples^17^ can be challenging.

In this work, we describe an interactive human-in-the-loop machine learning method enabling chemists to assign collections of molecules to multiple classes or clusters. The main goal of our method is to assist the human expert in the identification of potential structure–activity relationship (SAR) series from newly generated high-throughput screening (HTS) data. Our targeted application scenario is data exploration and grouping of molecules from HTS experiments based on molecular features that exploit the chemist’s expert knowledge. In the lead identification stage, grouping hits based on their structure and anticipated modes of action is an important preparatory step before subsequent SAR studies.^4^ The classification usually depends on the specific application and the chemist’s experience and preferences.^4^ The goal of this work is to enable a more effective and accelerated classification process. In contrast to earlier work,^4^ our new system is based on a constrained online support vector machine^22^ to iteratively integrate human feedback to solve the multiclass classification problem. To allow users to freely navigate and explore the data, we decided against the standard AL approaches in which the learning model selects which samples to label next.^2,4,9^ Therefore, the system presented belongs rather to the class of interactive machine learning methods than to AL.^2^ The vision is to put the chemist in control of the process, efficiently supported by the model. The concept is implemented in an exemplary, publicly accessible web server named PegaPlus with an interactive and feature-rich interface.

## Methods

### Overview

Our interactive machine learning method consists of three steps. An overview of our method is shown in Figure 1. Initially, an assignment of compounds into potential SAR series is generated using clustering based on molecular descriptors, e.g., circular fingerprints.^23^ This initial assignment is then refined interactively by the chemist, e.g., by reassigning a molecule from one cluster to another. The machine learning model is updated after each manual intervention using the user-given refinement and molecular descriptors. The model proposes a new overall assignment to the user for further refinement. This iterative cycle can be repeated until the user is satisfied with the resulting molecule clusters.

**Figure 1:**
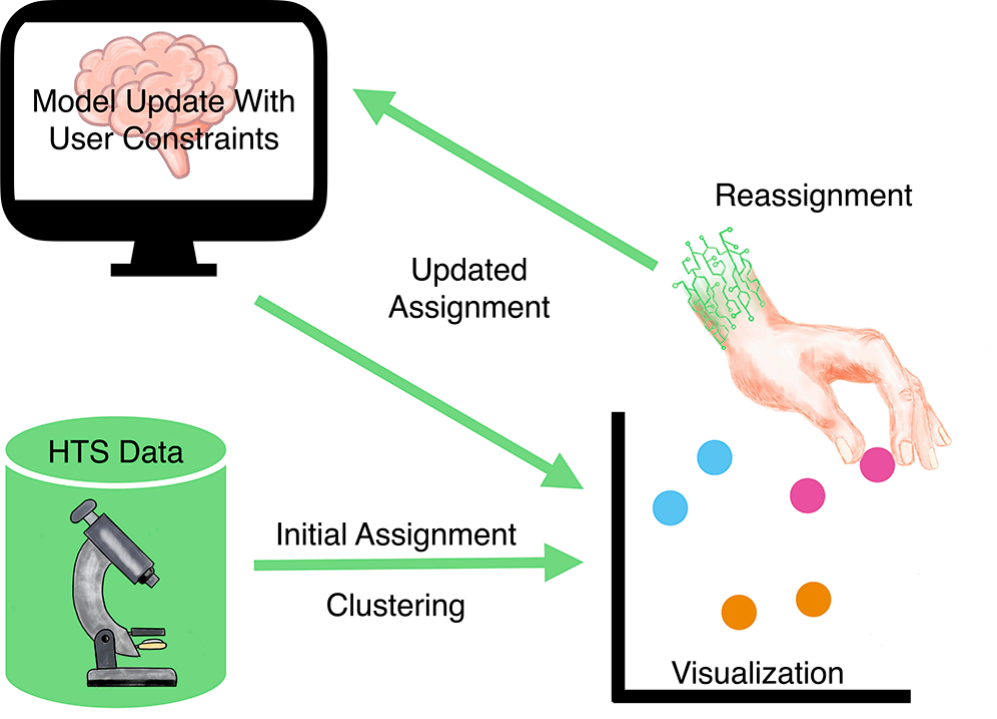
Overview of the PegaPlus method. After presenting an initial clustering, the reassignment phase starts, where molecules are manually reassigned by the user through the graphical user interface. This information is used to update the machine learning model before the updated result is again visualized. This reassignment and model update phase is repeated until the result appears appropriate to the user.

To showcase the PegaPlus concept, the process is implemented via a web service. In order to achieve interactivity, both the machine learning method and the visualization have to be very efficient.

In the following, we will first describe the initial clustering, followed by the embedding process. Next, the Online SVM is elaborated, and finally, the web server that visualizes the initial clustering, embedding, and outcome of the Online SVM algorithm is described.

### Initial Clustering

Clustering is an unsupervised machine learning method to discover inherent structures and patterns within unlabeled data. The k-means approach is one of the most popular clustering algorithms. Its main goal is to divide the data into *k* clusters, where each data point belongs to the cluster with the nearest mean based on feature similarity.^24^ The outcome of the standard k-means depends on the initial selection of cluster centers. To circumvent this limitation, the k-means++ implementation of sci-kit learn was used,^25–27^ which initializes cluster centers with an enhanced strategy. Initial centroids are selected using an empirical probability distribution of the points’ contribution to the overall inertia. In the sci-kit learn implementation, the best centroid from several sampling trials is selected.^25,26^

The RDKit Morgan Fingerprint with radius four, similar to ECFP8, was used as descriptor.^23,28^ The circular descriptor encodes local atomic environments within a molecule, subsequently folded to a fixed-length 1024-bit vector, capturing very detailed structural information.

The initial clustering is performed using the k-means++ algorithm with a user-defined parameter *k*. The clustering provides the foundation for the subsequent reassignment phase, during which *k* SVM models are trained.

### Embedding

In general, high-dimensional data is hard to interpret for humans. Embedding or dimensionality reduction methods can project high-dimensional data into low-dimensional space for visualization.^29^ We use embedding methods to reduce the high-dimensional descriptor space of the molecular HTS data and their cluster assignments to two dimensions. This is necessary to provide a tangible representation for an expert and to facilitate the visualization of the structure within the data.

An additional requirement for our application is that the embedding algorithm must be able to place individual data points (molecules) at coordinates according to the user’s specifications. This gives users more control over the exploratory process while accounting for the structure of data in high-dimensional space. The algorithm also needs to be fast enough for interactive use to provide the best possible user experience.

Many embedding algorithms, such as PCA (Principal Component Analysis),^30^ t-SNE (t-distributed stochastic neighbor embedding),^31^ and UMAP (Uniform Manifold Approximation and Projection)^32^ exist. While PCA is fast, it has disadvantages in mapping complex nonlinear patterns, and the coordinates are determined by the data itself and the PCA transformation. Conversely, t-SNE and UMAP are capable of visualizing complex nonlinear relationships and local clusters, but at the same time, the distances between points in the visualization are not directly interpretable as real distances in the original space. The t-SNE algorithm also needs more computation time than PCA.^31^

The Stochastic Proximity Embedding algorithm (SPE) is computationally efficient and can capture nonlinear relationships. Additionally, the algorithm works directly with the coordinates in the low-dimensional projection and modifies them based on the distances in the high-dimensional space. This allows the distances between the points to be interpreted as good approximations to the distances in high-dimensional space, which is a suitable property for our approach. Therefore, we decided to use SPE as our base method and modified it to meet the requirements of PegaPlus.

The native implementation of the SPE^33^ initially places data points, e.g., molecules, randomly in the low-dimensional space. Next, two data points *i* and *j* are randomly selected. The distance between the points in IR^*n*^ is compared to the corresponding one in low-dimensional embedding space. If these distances differ, a coordinate update process is performed, in which the coordinates of the data point *x*_*i*_ and *x*_*j*_ are updated by

1 $$x_{i}\leftarrow x_{i}+\lambda \frac{1}{2}\frac{r_{\mathrm{ij}}-d_{\mathrm{ij}}}{d_{\mathrm{ij}}+\varepsilon }\left(x_{i}-x_{j}\right)$$

and

2 $$x_{j}\leftarrow x_{j}+\lambda \frac{1}{2}\frac{r_{\mathrm{ij}}-d_{\mathrm{ij}}}{d_{\mathrm{ij}}+\varepsilon }\left(x_{j}-x_{i}\right)$$

where *d*_*ij*_ is the distance between data points *i* and *j* in low-dimensional space, and *r*_*ij*_ is the distance between them in high-dimensional space, λ is a learning rate parameter and ε is a small number to avoid a division by zero. By repeating the update process, the points in the low-dimensional space are arranged to minimize the difference in the pairwise distances of the data points between the high- and low-dimensional space. This achieves a self-organized embedding of molecules in low-dimensional space.

In our method, we want the embedding to obey user-given constraints. We call a data point constrained if it has been manually repositioned in low-dimensional space.

The native implementation of the SPE cannot handle constraints, but it is reasonably straightforward to modify the algorithm accordingly. By not updating the coordinates of constrained points, the self-organizing behavior of the algorithm should place the other molecules at the corresponding position in a low-dimensional space with respect to their distance in high-dimensional space. Effectively, user-constrained data points will serve as anchors for the other data points based on their position in low-dimensional space. To align data points to the constrained ones, the algorithm needs to select the constrained points frequently enough as coordinates in the update function. Since all other unconstrained data points must also be placed correctly in relation to each other, we added a heuristic to ensure that a constrained point for *x*_*i*_ is picked in 50% of the cases.

We generated examples representing typical rearrangements by a user to test that the adaptation to the SPE works as intended. Specifically, 1000 two-dimensional data points were synthetically generated using NumPy.^34^ The data points are grouped in two clusters, with one cluster in the lower left, *x* ∈[0, 0.4], *y* ∈[0, 0.4], and the other in the upper right, *x* ∈[0.6, 1], *y* ∈[0.6, 1], of the first quadrant. Based on these clustered data, user-defined constraints are simulated, and the resulting embeddings are examined, see Figure 2.

**Figure 2:**
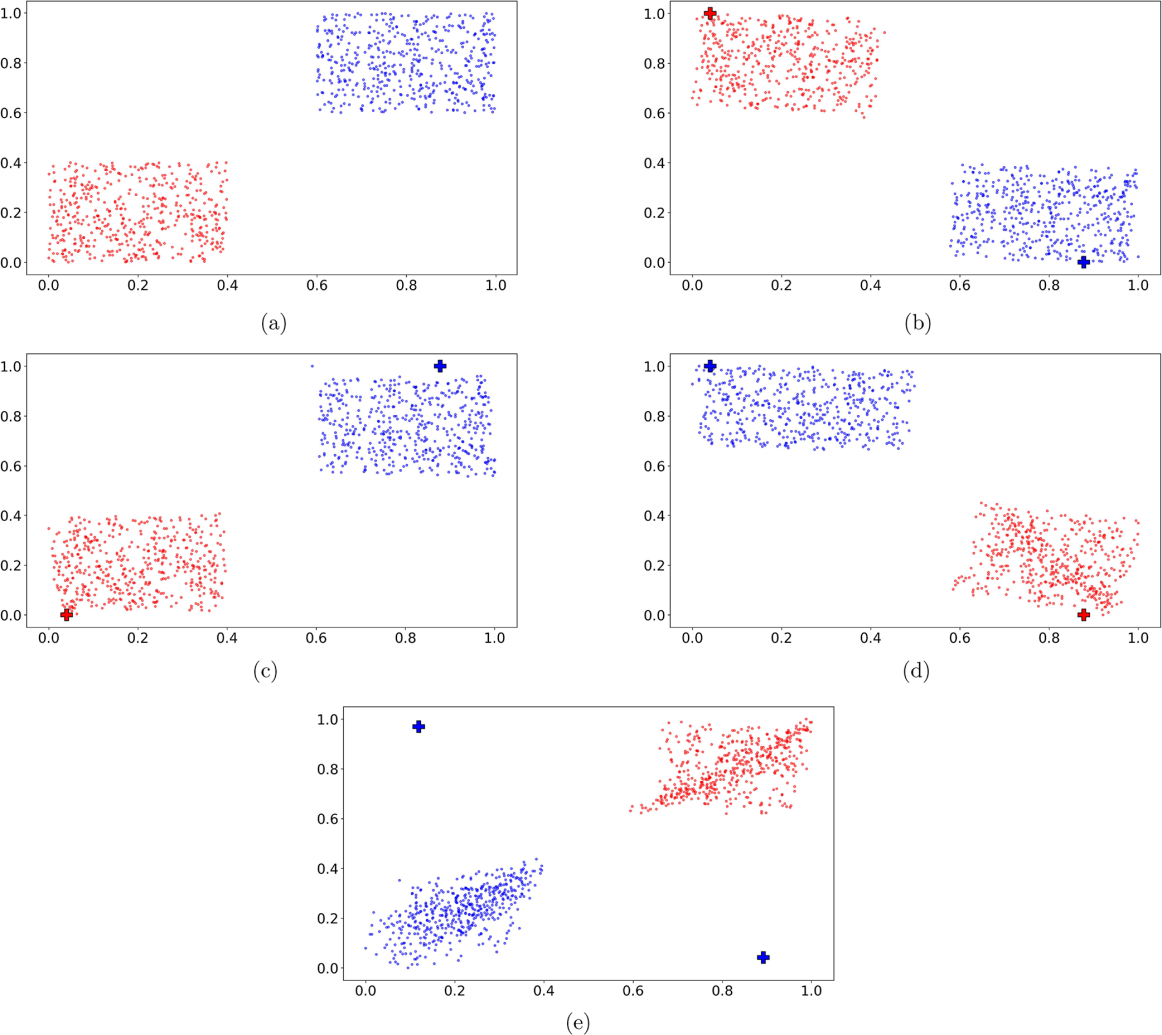
Evaluation results for the heuristic incorporating constraints into the SPE algorithm: synthetically generated data set with two clusters shown in red and blue (a), initial embedding results without constraints (b), embedding with two constraints (red and blue cross) with swapped *y* coordinates (c), embedding with two constraints with swapped *x* coordinates (d). (e) shows an embedding result where the previous constraints from (d) were released, and two nearby points from the blue cluster were repositioned farther apart as new constraints.

### Online SVM

As with embedding, a fast machine learning method capable of learning incrementally with single training points is required to ensure an interactive experience and incorporate specific learning constraints from user feedback.

Online learning^35^ is a suitable approach to incremental learning that allows models to be updated as new data become available. This allows continuous learning without the need for full retraining. Support Vector Machines (SVMs), among other machine learning methods, have been explored in the literature with these techniques.^35^ User feedback can be incorporated using active learning methods that have also been applied to SVMs.^4^ A support vector machine is a supervised machine learning algorithm used for classification and regression. During the learning process, SVMs identify the optimal hyperplane, which is the boundary separating data points of different classes with the maximum margin.^36^ The Pegasos (Primal Estimated sub-GrAdient SOlver for SVM) is a stochastic gradient descent method for solving the optimization problem posed by SVMs.^22^ Unlike standard SVM models, Pegasos models can be trained using single training examples. This is useful when working with user constraints that were not present during the initial learning process.

The Pegasos training procedure starts by selecting a random training example. The weight vector is then updated after each example. In case of misclassification, the weight vector at time *t* is updated as follows

3 $$w_{t+1}=\left(1-\eta_{t}\lambda \right)w_{t}+\eta_{t}y_{i_{t}}x_{i_{t}}$$

The weight vector is represented by **w**_*t*_ and $\eta_{t}=\frac{1}{\lambda t}$ . **x**_i_*t*__ is the feature vector of example *i*_*t*_ with label *y*_*i*_*t*__, and λ is the initial learning rate.

Unfortunately, this procedure does not natively work with constraints that are necessary for our approach. Learning a data point does not ensure that the hyperplane will change enough for the constraint to be classified correctly. There are multiple potential approaches for learning a user-provided constraint that has been misclassified. One approach is to repeat the update process until the example is correctly classified. However, this results in a remarkable increase in the number of computational steps that have to be applied. Another approach is to increase the learning rate to correct the prediction for the previously misclassified data point in a single step. However, if the learning rate value is not chosen carefully, this strategy could lead to dramatic overfitting with respect to a single data point. We have implemented a modified version of Pegasos to address iterative learning under user constraints with a focus on efficiency, enabling interactive use. We introduce a new weighting factor *c*_*i*_ to the previous equation and choose it for an incorrectly classified constrained data point (*x*_*i*_,*y*_*i*_) such that this point is guaranteed to be classified correctly and is not within the margins after the update, i.e., *y*_*i*_ ⟨**w**′, **x**_**i**_ ⟩ ≥ 1. See Section 2 Derivation of the new factor c of the Supporting Information for the derivation of the new weighting factor *c*_*i*_. This weighting factor *c*_*i*_ ensures that the weight vector, and therefore the decision boundary, is changed as little as possible so that the current constraint is correctly classified and lies outside the margin. The resulting algorithm produces similar results in less computation time compared to the standard algorithm, where a data point is repeatedly learned until it is correctly classified. The updated algorithm is shown in Algorithm 1. Please note that with our modification, only the most recent constraint is guaranteed to be classified correctly.
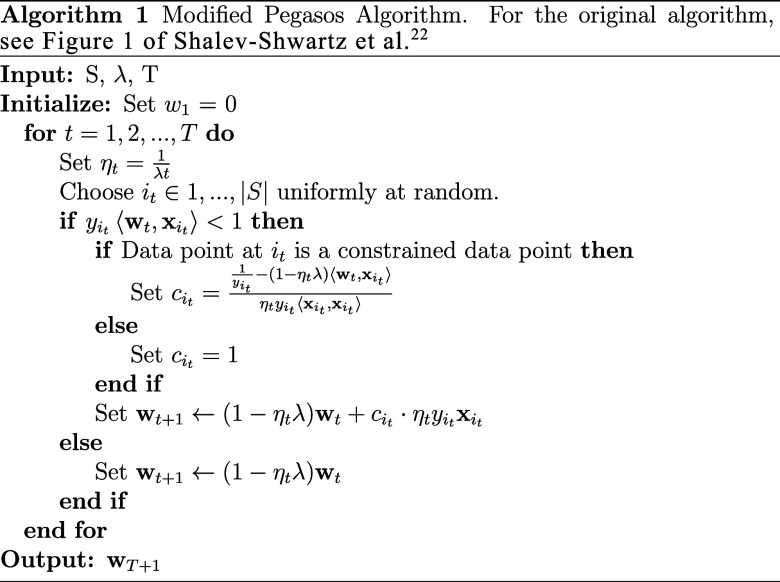


## Results

The evaluation results for the constrained SPE and the online SVM are shown below. After that, a description of the web server will be given.

### Embedding

Figure 2 shows the synthetic examples that test whether the proposed adaptations to the SPE algorithm work as intended. These examples should present typical rearrangements of molecule data points by a user in the 2D representation.

The original data is shown in Figure 2a and the initial embedding in Figure 2b. Our modifications did not affect the standard SPE embedding. The resulting embedding has a different rotation compared to the original data in Figure 2a. However, this is expected since the SPE algorithm is rotation-invariant.

We tested the modification introduced to the SPE to handle constraints in Figure 2c,d. Data points subject to constraints are highlighted by crosses of increased size. Figure 2c shows a second iteration of the modified SPE from the initial embedding in Figure 2b, but with specific constraints, indicated by the positions of the crosses. The red rectangular-shaped cluster has moved down, and the blue has moved up compared to the original. The nonconstrained data points follow the constrained points while preserving the cluster structure, demonstrating the desired behavior. Similarly, Figure 2d shows another constrained example, starting from the embedding shown in [Fig fig2]c. The result shows the blue and red clusters being flipped compared to the original embedding, putting the red cluster in the lower right corner and the blue cluster in the upper left. Again, the nonconstrained points follow the constrained points and successfully preserve the cluster structure during the self-organizing embedding procedure.

The algorithm should also be able to identify and visualize problems, for example, if the chosen descriptor does not contain relevant information. Figure 2e shows this experiment in which the constrained points, belonging to the same cluster (blue), are placed farther apart in opposite directions. The nonconstrained data points are realigned with respect to the two constraints and placed between the constrained points, while the red cluster is pushed into the remaining corner.

In all four embeddings, the clusters are separated from each other, as in the original visualization in Figure 2a. The experiments have shown that the constraints are sufficiently taken into account and that the changes do not negatively affect the actual algorithm. With these results, we are confident that the embedding meets the requirements and is suitable for the PegaPlus method.

### Online-SVM

In order to assess the efficacy of our modified Online-SVM, we utilize a manually annotated expert data set, originating from the DUD database.^37^ The compounds were clustered by Good et al. based on reduced graphs and manual inspection.^38^ The resulting data set is an ideal scenario, since the clustering does follow human intuition rather than strict algorithmic rules. Eight DUD targets were chosen, and these data sets were further processed as described by Lang et al.^4^ (i.e., removing small clusters). An overview of the resulting data sets is shown in Table 1. The P38 MAP, the AChE, and the PDGFrb data set were also used by Lang et al.^4^ for detailed analysis.

**Table 1:** Data Set Overview

data set	number of molecules	number of classes
P38 MAP	115	6
AChE	86	7
AR	56	3
COX-2	158	8
InhA	27	3
SRC	69	4
FGFr1	62	4
PDGFrb	109	10

To validate our approach and demonstrate a benefit when compared to the manual refinement, the machine learning process must be capable of producing the expert’s clustering, but with fewer iterations required. Starting from an initial clustering, the expert corrections have to be learned iteratively by the machine learning model. Since our approach combines an unsupervised and a supervised method, we have to propagate the supervision from the expert to ensure a comparable evaluation. In real applications, the expert chooses labels based on knowledge and experience. For evaluation, we match the labels of the initial clustering with the labels of the expert data set. To achieve this, the maximum matching between the initial clustering result and the expert result was calculated using the SciPy library.^39^ The maximum matching was then used to derive the target labels.

At first, learning of *k* models based on the initial clustering was performed using the Morgan Fingerprint mentioned above with radius four. We trained the models through a One-vs-All approach and set *k* to the number of clusters as in the expert clustering. Then, the expert’s manual reassignment process was simulated, and the models were updated after each reassignment iteratively. The Adjusted Rand Index^40,41^ was chosen as a metric to compare the learned clustering result to the expert result. To avoid typical oscillations of machine learning models between single data points after relearning, an Adjusted Rand Index of 0.95 was set as a stop criterion for achieving a satisfactory outcome. In order to keep the results comparable, the stop criterion applies to both machine learning and the manual approach.

Two distinct learning strategies have been explored. In the first learning strategy, the latest constraint and all previous constraints are learned. Previous constraints are only relearned when they are violated. In the following, this learning strategy is called All Constraints Learning Strategy. All constraints are shuffled before each learning. Subsequently, each constraint that was violated is learned again. The second strategy, termed the Single Constraint Learning Strategy, involves the learning of only the most recent constraint. With the manual postprocessing and the previously presented modification of the Pegasos algorithm, five experiments are carried out on each data set. The manual approach, the standard Pegasos with the All Constraints Learning Strategy, the standard Pegasos with the Single Constraint Learning Strategy, the modified Pegasos with the All Constraints Learning Strategy, and the modified Pegasos with the Single Constraint Learning Strategy. All experiments are repeated 1000 times. We used a paired sample *t*-test (calculated with SciPy^39^) to determine whether the mean difference of the number of user interventions between the manual approach and each machine learning-based learning strategy is zero. Each pair consists of the number of user interventions for the manual approach and the respective machine learning-based learning strategy for the same initial clustering.

As demonstrated in Figure 3, these findings indicate that, across all shown data sets, the All Constraints Learning Strategy consistently required a lower number of relearning iterations than the manual approach, except for the PDGFrb data set, where comparable results were achieved. The results from the other data sets can be found in Figure S1 of the Supporting Information and support this observation. Utilizing the Single Constraint Learning Strategy, an improvement is visible compared to the manual adaptations in four of the eight test cases. We used the Morgan Fingerprint with radius four which encodes a lot of information. By reducing the radius, less information will be encoded, which also has a slightly negative effect on the performance of our approach, as shown in Figure S2 in the Supporting Information. Since the All Constraints Learning Strategy showed a consistent improvement with respect to the number of iterations, it became our preferred strategy.

**Figure 3:**
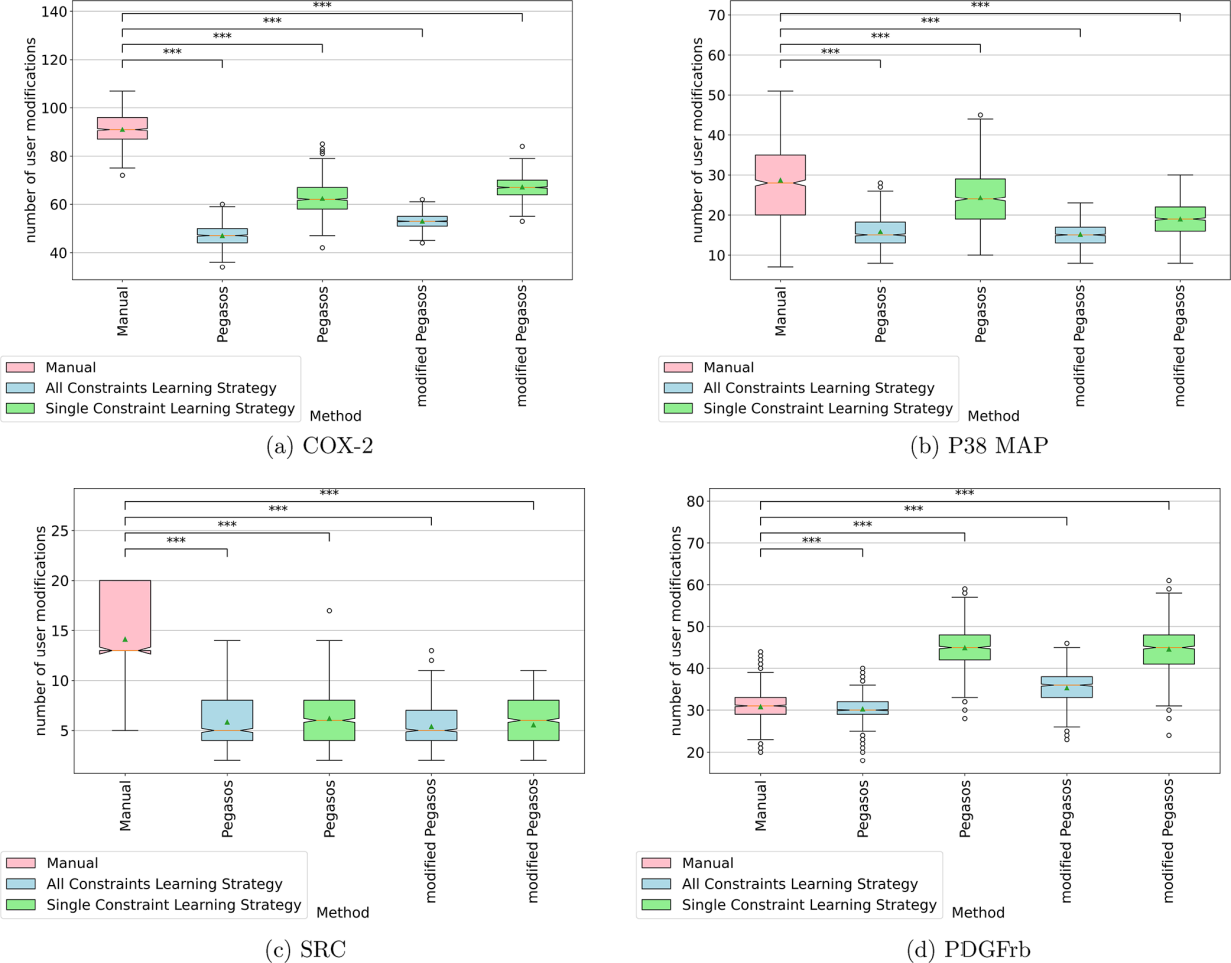
Box plots for five combinations of iterative learning strategies and the Pegasos algorithm (averaged over 1000 repetitions). The *x*-axis displays the different approaches, and the *y*-axis displays the number of user modifications required to achieve a satisfactory result (Adjusted Rand Index >0.95). Significance brackets indicate the significance of the differences of the number of user modifications of the ML-based learning strategies relative to the manual approach (paired sample *t*-test) with *** indicating an uncorrected *p*-value <0.001. The red boxes represent the manual approach, the blue boxes represent the all constraints learning approach, and the green boxes represent the single constraint learning approach. For the latter two, the first box corresponds to the standard Pegasos algorithm and the second to the modified version. Results are shown for the targets (a) cyclooxygenase-2, (b) P38 mitogen activated protein, (c) tyrosine kinase SRC, (d) platelet derived growth factor receptor kinase.

As shown in Figure 3, the modification made to the Pegasos algorithm shows comparable results to the standard version, but the number of computational steps is significantly reduced, as shown in Figure 4. It should be noted that all model updates are taken into account. If a data set has three classes, then three models exist, which have to be updated for a single constraint. We also used a paired sample *t*-test (calculated with SciPy^39^) to determine for both constraint learning strategies whether the mean difference of the number of learning (i.e., computational) steps between the standard and the modified Pegasos algorithm is zero. Each pair consists of the number of learning steps for the standard and the modified Pegasos algorithm for the same initial clustering.

**Figure 4:**
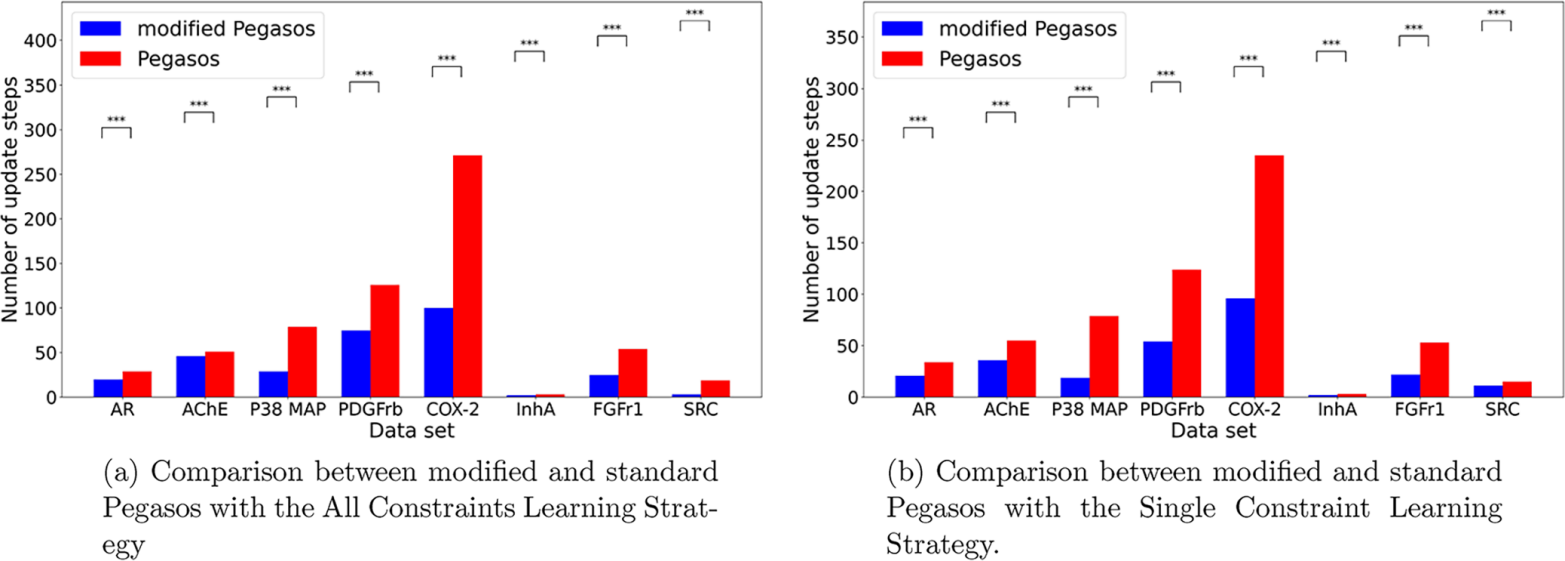
Bar plot showing the mean number of update steps required to train the models to achieve an Adjusted Rand Index of 0.95, averaged over 1000 repetitions. For each data set, the red bars represent the standard Pegasos algorithm and the blue bars represent the modified version. The number of update steps refers to the number of times the models were updated. Significance brackets indicate the significance of the differences of the number of learning steps of the modified and the standard Pegasos algorithm (paired sample *t*-test) with *** indicating an uncorrected p-value <0.001.

The maximum runtime across all experiments was 325.26 ms for the COX-2 data set when using the Pegasos method with the Single Constraint Learning Strategy. Since a complete experiment on the data sets could be completed in less than a second in the worst-case scenario, we are convinced that the runtime is short enough to enable interactive work. The runtime for all experiments can be found in the Supporting Information, Section 5: Runtime results. All experiments were performed on a standard 2023 MacBook Pro with an M2 Max processor and 96 GB of memory, running single-threaded.

For the InhA data set, the Adjusted Rand Index starts at a value of 0.876 in 80% of the experiments. After one adjustment, the value exceeded 0.95, which is the stopping criterion for a satisfactory outcome.

As shown in Figure 3d, the Pegasos approach performs only slightly better than the manual approach on the PDGFrb data set. Figure 5 compares the intra- and intercluster distances of individual molecules based on Morgan fingerprints with a radius of four. The silhouette *s* quantifies how well each molecule fits its assigned cluster relative to the nearest alternative cluster. The diagonal represents *s* = 0. Molecules below the diagonal (*s* < 0) are more similar to a neighboring cluster than to their assigned cluster. This applies to 17 molecules of various clusters in the PDGFrb data set. By contrast, the SRC data set contains only six such molecules, all of which are from the same cluster. This could explain why our approach requires a similar number of steps as the manual approach for the PDGFrb data set and yet outperforms the manual approach for the SRC data set. Plots for all data sets can be found in the Supporting Information, Section 6 Intra- and intercluster comparison.

**Figure 5:**
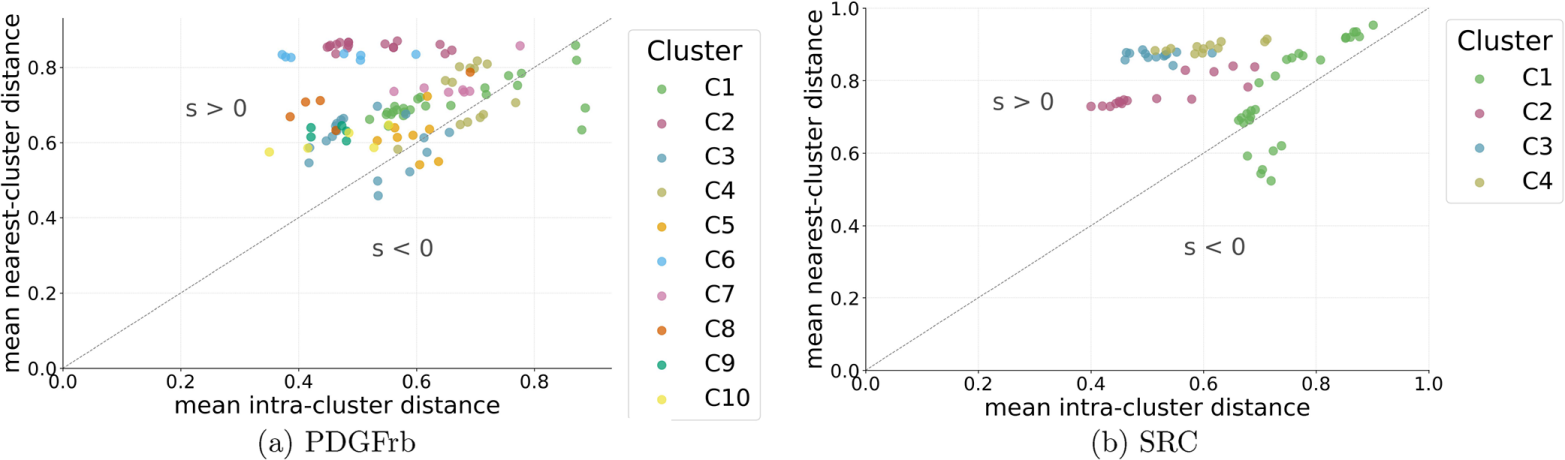
Scatter plot of mean intracluster distance versus mean nearest-cluster distance for the SRC and PDGFrb data sets. Each data point represents a molecule, colored by the cluster assignment of the expert clustering. Distances are computed as 1 - Tanimoto similarity^42^ using Morgan fingerprints with radius 4 and 1024 bits. A silhouette of *s* < 0 indicates that molecules are closer to a neighboring cluster than to their own.

The complexity of the PDGFrb data set is also visible in Figure 6. It should be noted that while both approaches are based on the same initial k-means++ clustering, the starting points of the Adjusted Rand Index can differ. This is because the machine learning models are first trained before the initial Adjusted Rand Index is calculated. Furthermore, once a run reaches a final Adjusted Rand Index of >0.95, the last observation is carried forward to avoid misleading drops caused by completed runs. Starting with an Adjusted Rand Index of 0.51 from the k-means++ clustering for PDGFrb, the active learning approach initially yields lower values for the Adjusted Rand Index than the manual approach. It is only after an average of 30 iterations that the manual approach is surpassed. In contrast, for the SRC and P38 MAP data sets, which start with similar values of 0.56 and 0.52 respectively, the active learning approach consistently outperforms the manual approach. The most significant improvement is seen in the COX-2 data set, where active learning starts with an initial Adjusted Rand Index of 0.06 and reaches 0.95 after approximately 46 iterations, whereas the manual approach requires 91 iterations. Without any interaction, k-means++ alone yields Adjusted Rand Index values between 0.05 and 0.86 across all eight data sets, yet both approaches consistently achieve a final Adjusted Rand Index of over 0.95. The active learning approaches require on average 30% fewer iterations, highlighting the contribution of the active learning as opposed to the a simple clustering. The convergence behavior across all experiments can be found in the Supporting Information, Section 7.

**Figure 6:**
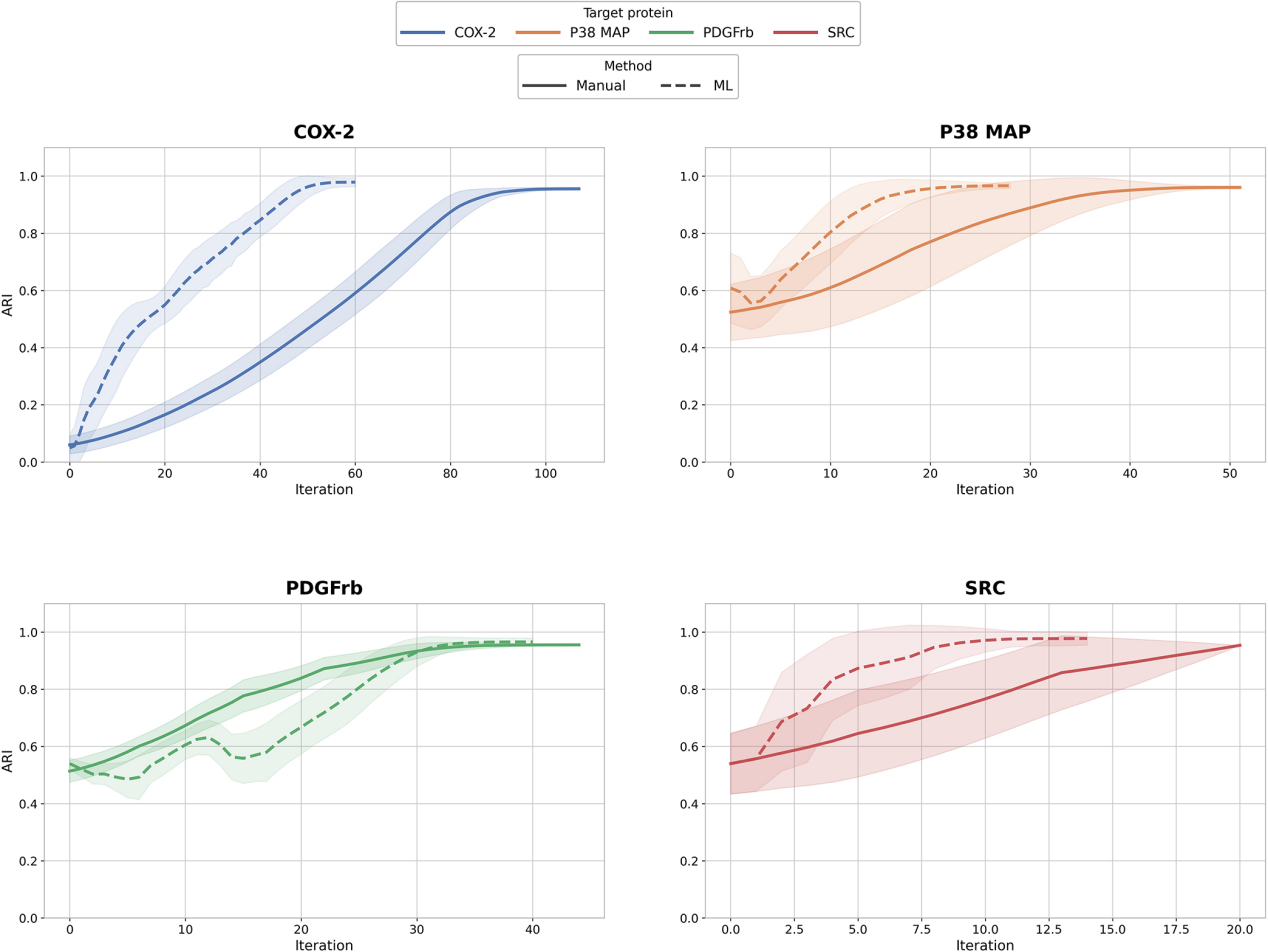
Progression of the Adjusted Rand Index for the experiments using Morgan Fingerprints with a radius of four and 1000 repetitions. Results are shown for the All Constraints Learning Strategy and Pegasos. The bold lines represent the mean Adjusted Rand Index, and the shaded areas indicate the standard deviation. The solid lines represent the manual approach and the dashed lines the machine learning approach. Results are shown for the targets cyclooxygenase-2, P38 mitogen activated protein, platelet derived growth factor receptor kinase and tyrosine kinase SRC.

The experiments have shown that both algorithms in combination result in an effective solution for compound clustering with manual intervention. The unmodified Pegasos outperforms the manual approach for every data set, while the modified Pegasos outperforms the manual approach for all data sets except one, for which comparable results are achieved. Furthermore, the modified Pegasos algorithm greatly reduces the number of required update steps. We are therefore confident that both algorithms meet the requirements for interactive processing and are suitable for a web application.

### Web Server

PegaPlus implements the methods introduced above to facilitate the exploration of new data sets. The web server, written with Django^43^ and vue.js,^44^ is intended to showcase the algorithms and the workflow in general rather than presenting a full-fledged tool for HTS data analysis. The calculation of chemical properties and descriptors relies on RDKit.^28^ The server workflow is initiated by uploading a data set in SDF format, selecting descriptors for machine learning, and the machine learning methods together with a few hyperparameters. For visualization purposes, it is possible to incorporate activity data into the SDF by annotating the data with an SDF tag designated as activity_value. After submission, the server performs the initial learning process, which includes clustering and the first round of training. Then, it displays the results based on the previously described algorithms. As illustrated in Figure 7, the molecules are embedded into a drawing plane and colorized by their cluster on the left side. Within this cluster view, molecules can be freely moved and assigned to a new cluster. Additionally, the user can delete and create new clusters as well as change the coloring scheme of the displayed data based on the activity of the analyzed molecules. To visualize the molecule in a 2D picture, we used SMILESDrawer.^45^ A tooltip with the respective molecular structure appears when the cursor hovers over a specific data point on the drawing plane. To facilitate a more comprehensive visual examination, a grid view module has been implemented. By clicking on a molecule in the grid view, the respective molecule is highlighted in the drawing plane. To further facilitate the exploratory process, a filtering mechanism has been incorporated to visually filter the molecules based on various descriptors, such as calculated logP by the method of Wildman and Crippen^46^ and heavy atom molecular weight. Additionally, a correlation and a distribution plot of molecular properties can be displayed to gain a deeper understanding of the data set, as shown in Figure 8. After each modification, a new learning calculation can be initiated to obtain an updated version where all new constraints are considered. At any point, it is possible to download the result or delete the entire project.

**Figure 7:**
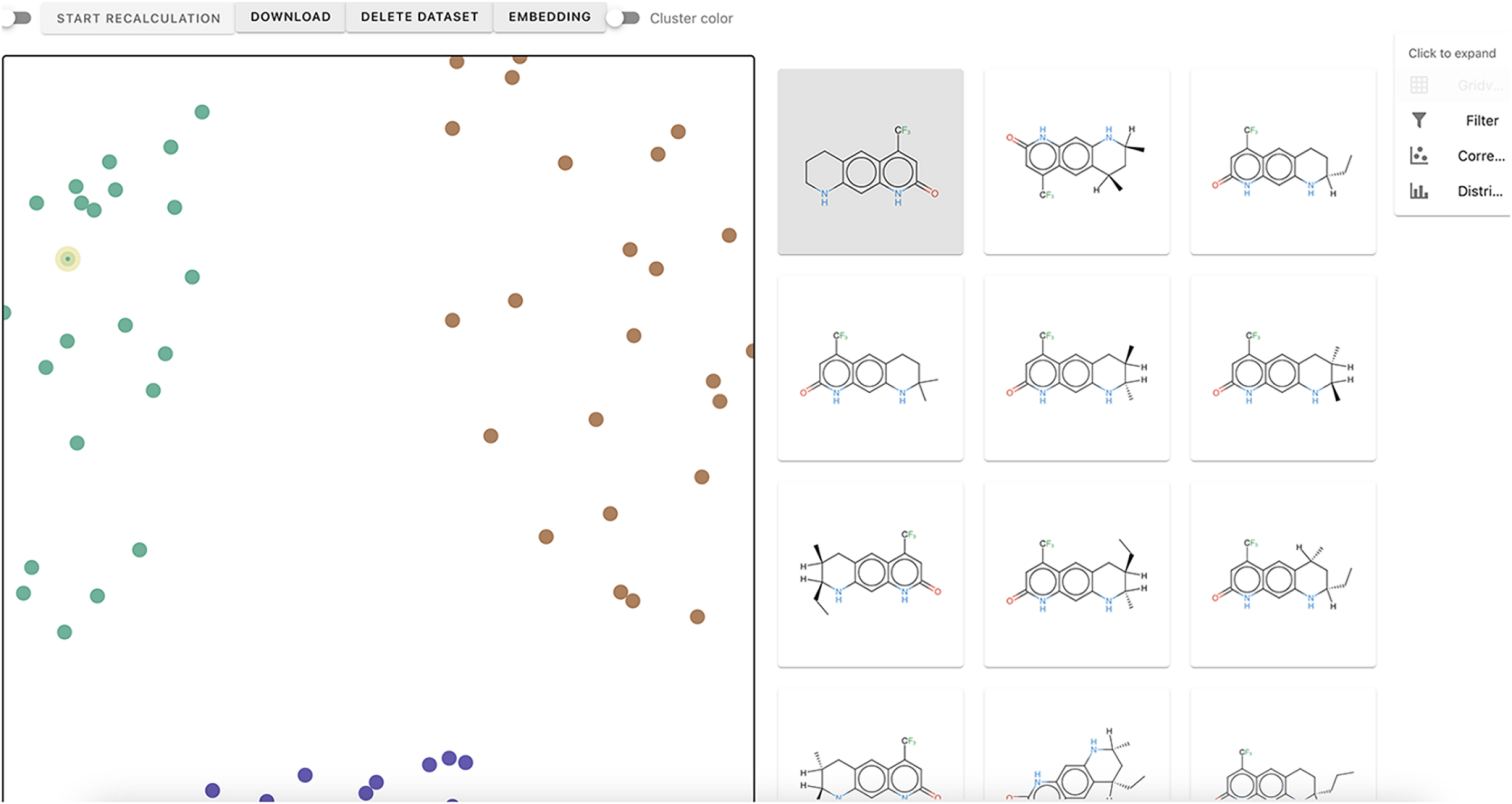
Interface of the PegaPlus web server. Convenient data analysis is enabled by graphical elements like a grid view of the molecules and multiple options for data visualization. The molecule selected in the grid view is highlighted as a yellow pulsating dot in the 2D plane (upper left corner).

**Figure 8:**
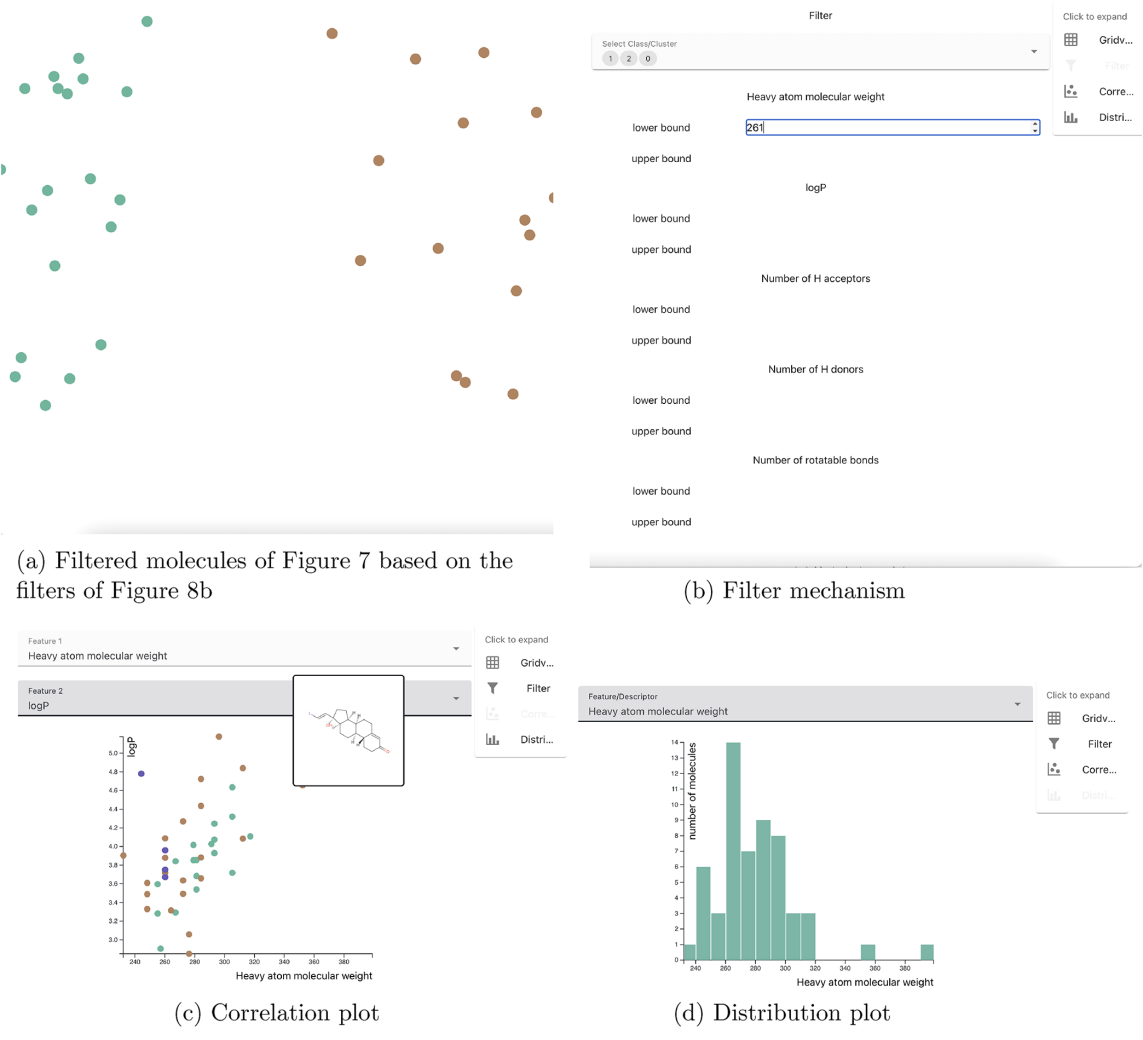
Components of the PegaPlus web server. (a) and (b) display the filter mechanism. There are currently six filters: heavy atom molecular weight, log*P*, number of hydrogen bond acceptors, number of hydrogen bond donors, and number of rotatable bonds. (c, d) show the correlation and distribution plots, respectively. The same properties can be set as in the filter mechanism.

To demonstrate how the methods work together within the web server, we conducted a simulation aimed at achieving the expert clustering of Good et al.,^38^ starting from an initial clustering of the SRC data set. Five recalculations were necessary in total, with Figures 9 and 10 showing the first two and next three iterations, respectively. Throughout the experiment, the yellow and blue clusters remained stable and consistent with the expert clustering. However, the molecules in the green and magenta clusters needed to be reassigned iteratively through user-provided constraints to align with the expert’s results. This is consistent with the results shown in the scatter plot in Figure 5. Nevertheless, all data points ultimately form distinct clusters, as shown in Figure 10d.

**Figure 9:**
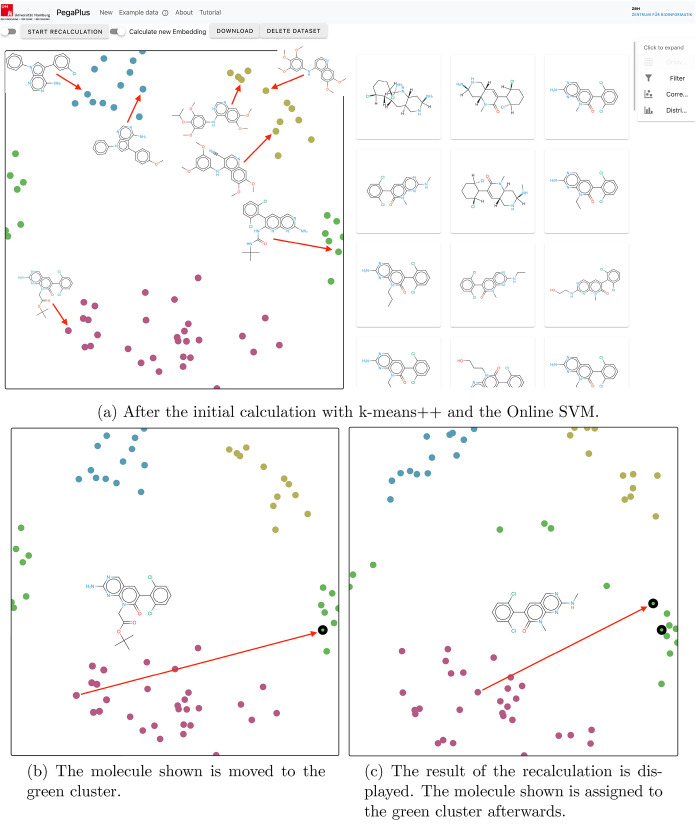
Initial processing of the SRC data set using the web server. Molecules are displayed as colored circles on the drawing plane, with their respective cluster colors. Constraints are displayed with thick black borders. (a) shows the full interface. (b, c) only show the drawing plane. The red arrows in (b, c) indicate motion. In (a), the arrows merely indicate the position of the corresponding molecular structure. The circle at the beginning of the arrows in (b, c) shows the initial position of the molecule, and the circle at the end shows its new position before recalculation. The cluster colors of the molecules are the same as in Figure 5.

**Figure 10:**
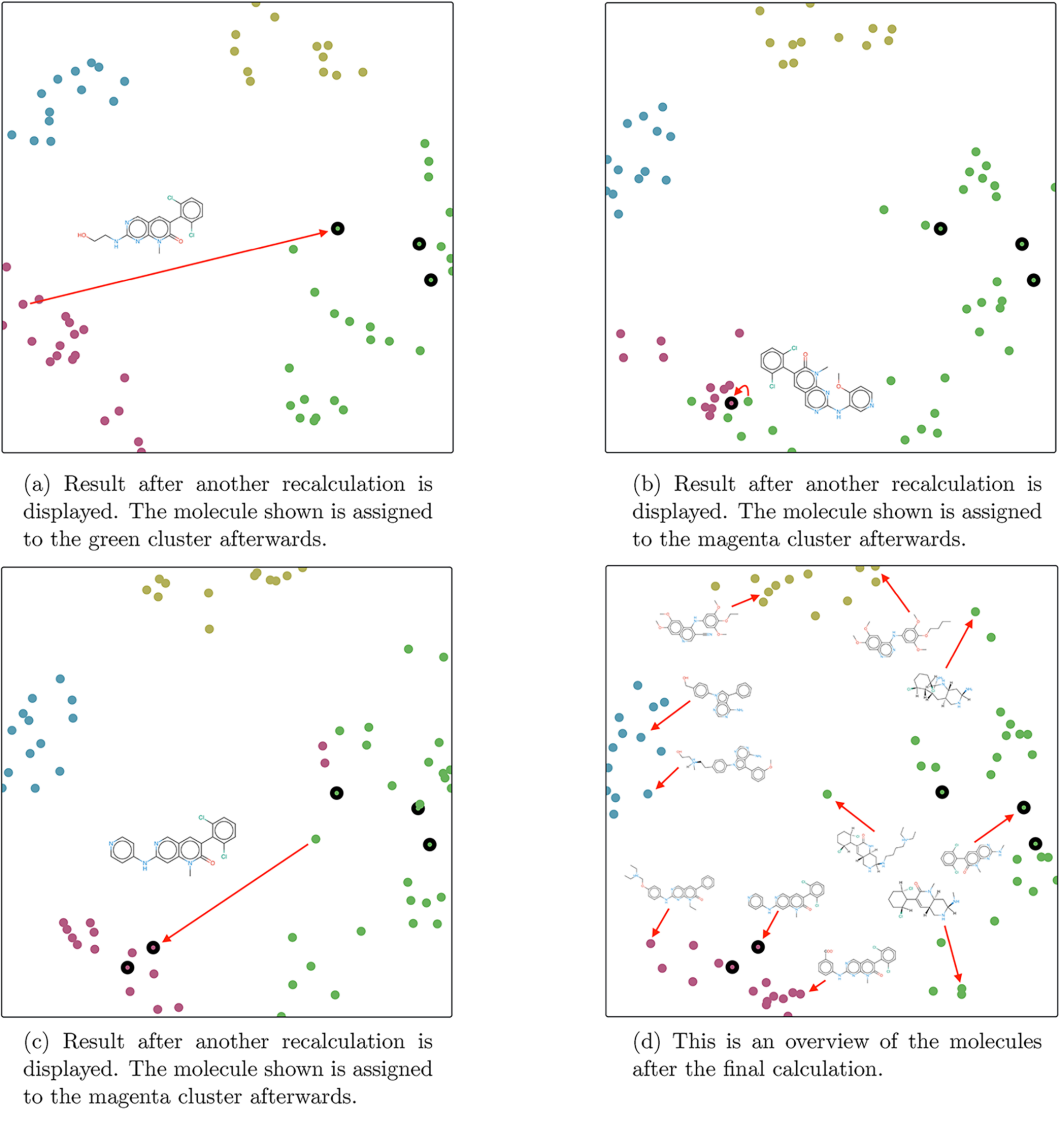
Continued processing of the SRC data set using the web server. Molecules are displayed as colored circles on the drawing plane, with their respective cluster colors. Constraints are displayed with thick black borders. (a)–(d) only show the drawing plane. The red arrows in (a)–(c) indicate motion. In (d), the arrows merely indicate the position of the corresponding molecular structure. The circle at the beginning of the arrows in (a)–(c) shows the initial position of the molecule, and the circle at the end shows its new position before recalculation. The cluster colors of the molecules are the same as in Figure 5.

An interesting case is shown in the embedding in Figure 10b which revealed a difference between the ground truth expert clustering and the calculated clustering. The discrepancy becomes visually apparent through the close proximity of differently colored points. Therefore, a constraint was added and the clustering was recalculated, leading to the result shown in Figure 10c. Here, two molecules from the magenta cluster were embedded near the green cluster. Upon closer examination of the surroundings of these two molecules, a third molecule with a different cluster assignment compared to the expert clustering can be observed and correctly reassigned. The ability to visualize and detect such molecules with undesirable cluster assignments that would otherwise go unnoticed in a purely automated process demonstrates the effectiveness of our combined approach of active learning and interactive visualization.

Since PegaPlus allows users to add or remove clusters, an additional simulation was conducted with an incorrect number of clusters, i.e., two instead of four. In contrast to the standard simulation, in which the yellow and blue clusters remained stable from the beginning, the two-cluster initialization fully merged these 25 molecules with the other clusters. This required substantially more constraints to recover the correct clusters, explaining the increase from an average of five to 17 constraints. The average number of five constraints has been depicted in Figure 3. Nevertheless, the correct clustering was recovered, demonstrating the robustness of the approach even under suboptimal initialization conditions. Details are provided in Section 9 of the Supporting Information. During our experiments with the SRC data set, we observed that the embedding heuristic performs better when more constraints are included, for example when constraints are available for all clusters. This observation is not surprising, as the algorithm then has access to additional information, including the expected number of clusters, which is typically an unknown variable in clustering applications. These findings further highlight the synergy between important expert knowledge and active learning. Whether this behavior generalizes beyond the present setting would require a larger evaluation study. The complete workflow, including additional molecular structures, is provided in Section 8 of the Supporting Information in the Web server experiment using the SRC data set.

## Conclusion

This publication focuses on advanced methods required for interactive compound clustering. The PegaPlus approach has the exploratory data analysis workflow of the medicinal chemist expert in mind. Integrated in a user-friendly web interface, the proposed methods enable online learning based on manual decisions. Since clustering is notoriously ill-defined, a semiautomated, user-guided process offers an effective balance between output quality and efficiency. The user interface is implemented through the PegaPlus web server, which is designed to assist medicinal chemists. The interactive nature requires careful coordination between the user and the learning algorithm to generate chemically intuitive clusters and find decisive molecules for the model to learn. The functions of the PegaPlus, such as filters and plotting mechanisms, are essential tools to achieve this goal. The server has been developed with software extensibility in mind, allowing for the addition of new features and functionality as required. So the filters could be expanded in the future to further enhance the user experience. The same applies to the machine learning methods. However, they must fulfill some requirements: be online-capable, allow multiple classes, and have constraint support.

At present, our approach offers one method (k-means++), which requires the specification of the number of clusters, for initial clustering. With an unknown data set, it is difficult to provide this information in advance. However, our web server offers the possibility to manually create new clusters or to combine two into one. Consequently, it is not necessary to restart the initial clustering to adapt the number of clusters. Furthermore, additional clustering algorithms could be implemented and integrated.

For visual guidance, we used an embedding algorithm with constraint support to display the molecules in a 2D plane, enabling users to arrange them as they wish. The experiments have shown that the constraints are sufficiently taken into account and that the changes do not negatively affect the actual algorithm. The algorithm organizes the molecules according to their structure in high-dimensional space and allows some manual adaptation to the nature of the data set.

In our experiments, all features are considered equally during the embedding calculation. Together with the weight vector of the SVM, corresponding features can be weighted in order to achieve a different embedding. This feature was also added to our PegaPlus server, but whether this embedding works better and reflects the chemist’s intuition better has to be shown on real-life data sets in the future.

Our learning experiments have shown that the algorithms can achieve the desired result within an equal or lower number of steps than manual correction. Here, the user was only simulated by always inserting the first misclassified molecule into the relearning process. However, users can explore the data set in different ways. Therefore, it would also be interesting to simulate other user behaviors when evaluating our method. Here, empirical studies with users could help to identify other data exploration strategies.

Our modification of the Pegasos SVM resulted in a significant reduction of the number of update steps in some cases, but it was not possible to conclude from our experiments whether our modified version could perform better on the classification task than the original one. Here, an analysis of larger data sets could provide new insights.

Future evaluations could investigate further descriptors besides structural fingerprints, for example, physicochemical features^47^ or learned representations, like neural fingerprints.^48^ Other potential future extensions could include a visual overview of all chemical structures or a visualization for a major fraction of a cluster by using Bemis-Murcko scaffolds,^49^ the maximum common substructure,^50^ and other highlighting of relevant substructures. Our PegaPlus approach combines interactive machine learning methods with additional filtering and plotting mechanisms in an interactive web server. We believe that this hybrid approach, combining manual decisions with online machine learning, is ideal for HTS data analysis, leading to accelerated processes and hopefully new insights.

## Supplementary Material



## Data Availability

The PegaPlus web server is available at pegaplus.zbh.uni-hamburg.de. A Jupyter notebook for the embedding experiments performed in this paper is available at https://github.com/rareylab/pegaplus/. Python scripts for the online SVM experiments are available at https://github.com/rareylab/pegaplus/, along with the data sets used during the experiments.

## Notes

The derivation of the new factor *c* introduced in Section Online SVM, the results for the remaining data sets used for validating our approach, the results of the evaluation experiments using Morgan fingerprints with a radius of two instead of four, the runtime results, the full results of the intra- and intercluster comparison, the Adjusted Rand Index convergence behavior of Pegasos and modified Pegasos across the Online SVM experiments and additional information about the simulation web server experiments (PDF)

## ‡

BASF SE, Ludwigshafen 67056, Germany

## §

BioSolveIT GmbH, An der Ziegelei 79, 53757 Sankt Augustin, Germany
