# Supplementary material for: PegaPlus—Interactive Machine Learning by Human Observation for Efficient Clustering and Analysis of Structure–Activity Data
Source: J Chem Inf Model. 2026 Jun 23;66(13):7501–14. doi: 10.1021/acs.jcim.6c00524 (PMC13370860; doi:10.1021/acs.jcim.6c00524)
Supplement: Supplementary file 1 [file ci6c00524_si_001.pdf]

Supporting Information: PegaPlus - Interactive Machine Learning by Human Observation for Efficient Clustering and Analysis of Structure-Activity Data

Rainer Fährrolfes,[†] Jochen Sieg,[†,‡] Florian Flachsenberg,[†,¶] and Matthias Rarey[*,†]

†*University of Hamburg, ZBH - Center for Bioinformatics, Albert Einstein Ring 8-10, 22761 Hamburg, Germany*

‡*Current address: BASF SE, Ludwigshafen, 67056, Germany*

¶*Current address: BioSolveIT GmbH, An der Ziegelei 79, 53757 Sankt Augustin, Germany*

E-mail: matthias.rarey@uni-hamburg.de

1 Additional resources

We provide additional resources for data and information. First, we derive the new factor c introduced in Section Online SVM in the main paper. Second, we present the remaining results of experiments that evaluated our approach. Third, we present the results of experiments that evaluated our approach using Morgan fingerprints with a radius of two instead of four. Fourth, we provide an overview of the distribution of runtime measurements for all runs. Fifth, we present plots showing the relationship between the mean intra-cluster distance and the mean nearest-cluster distance for all data sets. Sixth, we show an overview

of how the Adjusted Rand Index changed across all experiments. Seventh, we present a simulation from the web server experiment using the SRC data set. Lastly, we present a simulation from the web server experiment using the same data set but with an incorrect number of clusters.

2 Derivation of the new factor c

$$y_i \langle \mathbf{w}', \mathbf{x_i} \rangle \geq 1 \tag{1}$$

$$y_i \langle ((1-\eta\lambda)\mathbf{w} + c\eta y_i \mathbf{x_i}), \mathbf{x_i} \rangle \geq 1 \tag{2}$$

$$(1-\eta\lambda)\langle \mathbf{w}, \mathbf{x_i} \rangle + c\eta y_i \langle \mathbf{x_i}, \mathbf{x_i} \rangle \geq \frac{1}{y_i} \tag{3}$$

$$c\eta y_i \langle \mathbf{x_i}, \mathbf{x_i} \rangle \geq \frac{1}{y_i} - (1-\eta\lambda)\langle \mathbf{w}, \mathbf{x_i} \rangle \tag{4}$$

$$c \geq \frac{\frac{1}{y_i} - (1-\eta\lambda)\langle \mathbf{w}, \mathbf{x_i} \rangle}{\eta y_i \langle \mathbf{x_i}, \mathbf{x_i} \rangle} \tag{5}$$

Set $c = \frac{\frac{1}{y_i} - (1-\eta\lambda)\langle \mathbf{w}, \mathbf{x_i} \rangle}{\eta y_i \langle \mathbf{x_i}, \mathbf{x_i} \rangle}$

3 Additional results experiment

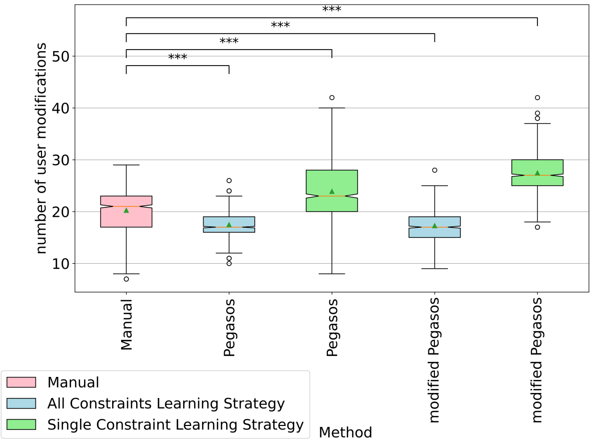

(a) AChE

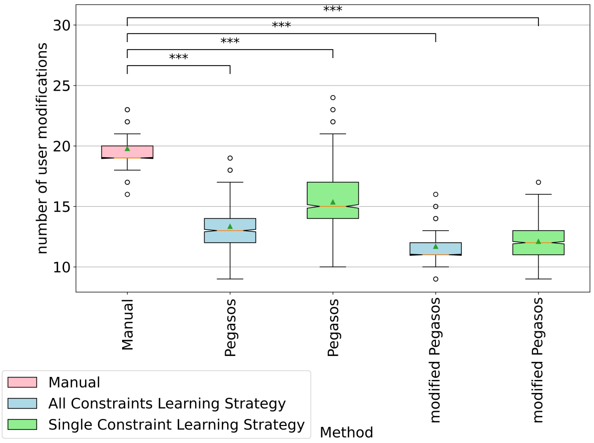

(b) AR

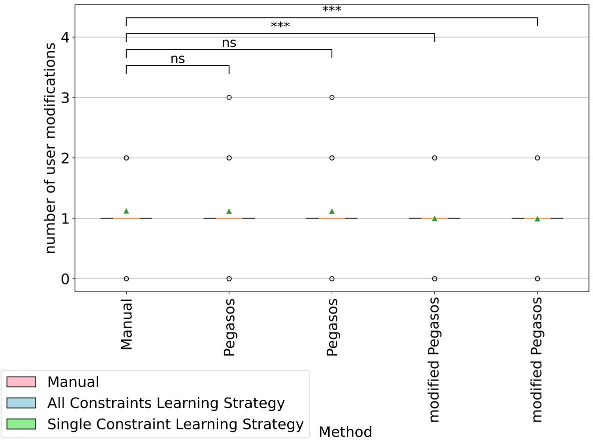

(c) InhA

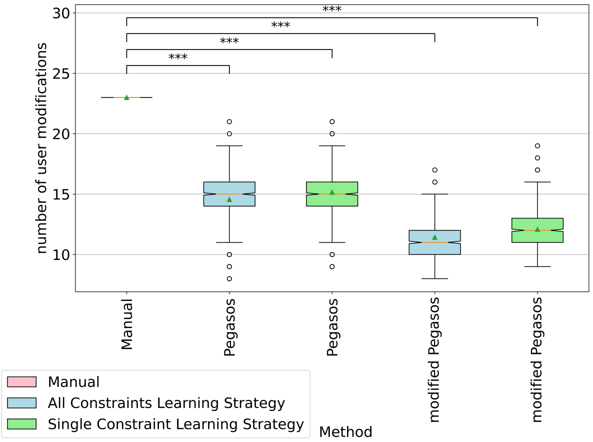

(d) FGFr1

Figure S1: Box plots for five combinations of iterative learning strategies and the Pegasos algorithm (averaged over 1000 repetitions). The x-axis displays the different approaches, and the y-axis displays the number of user modifications required to achieve a satisfactory result (Adjusted Rand Index > 0.95). Significance brackets indicate the significance of the differences of the number of user modifications of the ML-based learning strategies relative to the manual approach (paired sample t-test) with *** indicating an uncorrected p-value < 0.001 and and ns indicating not significant. The red boxes represent the manual approach, the blue boxes represent the all constraints learning approach, and the green boxes represent the single constraint learning approach. For the latter two, the first box corresponds to the standard Pegasos algorithm and the second to the modified version. Results are shown for the targets (a) acetylcholinesterase, (b) androgen receptor, (c) enoyl ACP reductase, (d) fibroblast growth factor receptor kinase.

4 Morgan fingerprint with radius two

Figure S2 shows the results when the Morgan fingerprint with radius two is used as a descriptor for the learning task. Figure S3 shows the number of times the models were updated. It should be noted that all model updates are taken into account. If a data set has three classes, also three models exist, which have to be updated for a single constraint. Here, also the Morgan fingerprint descriptor with radius two was used.

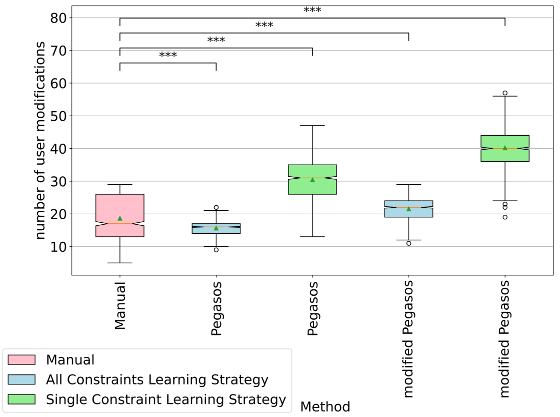

(a) AChE

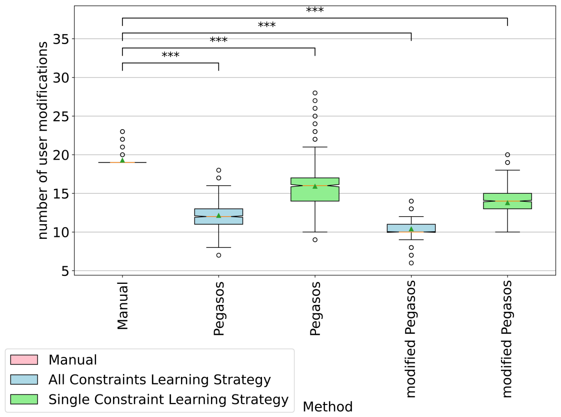

(b) AR

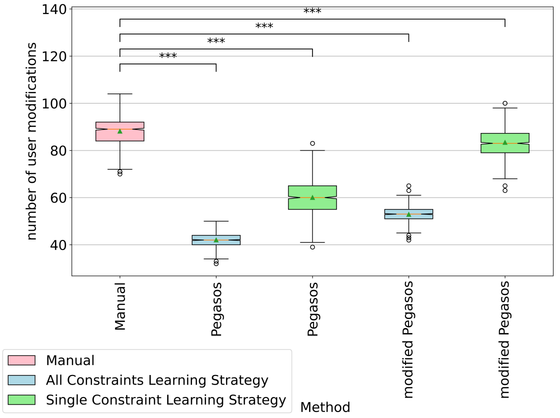

(c) COX-2

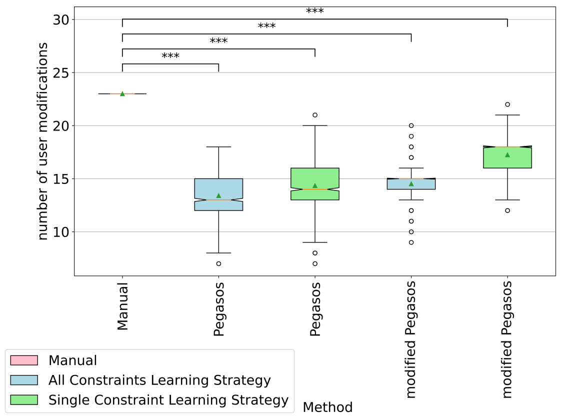

(d) FGFr1

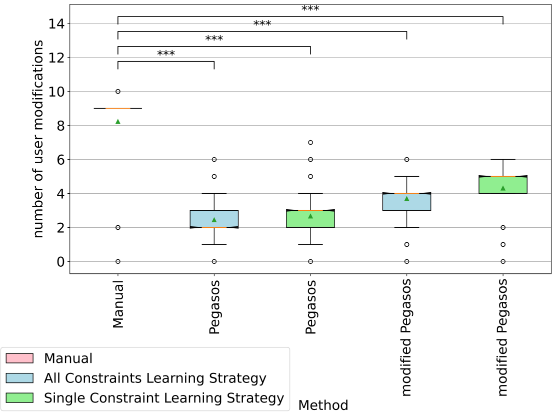

(e) InhA

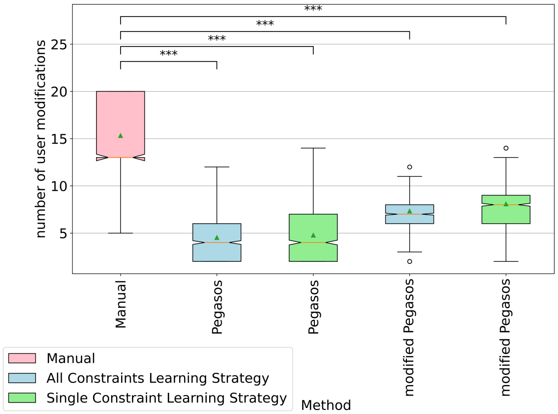

(f) SRC

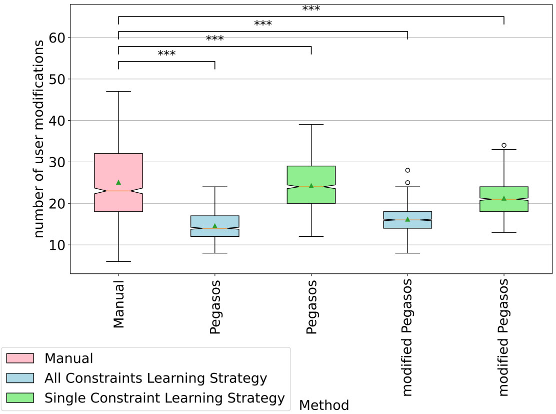

(g) P38 MAP

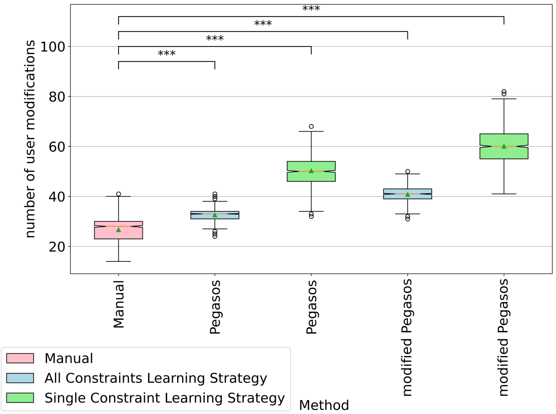

(h) PDGFrb

Figure S2: Box plots for five combinations of iterative learning strategies and the Pegasos algorithm (averaged over 1000 repetitions) using Morgan Fingerprint with radius 2. The x-axis displays the different approaches, and the y-axis displays the number of user modifications required to achieve a satisfactory result (Adjusted Rand Index > 0.95). Significance brackets indicate the significance of the differences of the number of user modifications of the ML-based learning strategies relative to the manual approach (paired sample t-test) with *** indicating an uncorrected p-value < 0.001. The red boxes represent the manual approach, the blue boxes represent the all constraints learning approach, and the green boxes represent the single constraint learning approach. For the latter two, the first box corresponds to the standard Pegasos algorithm and the second to the modified version. Results are shown for the targets (a) acetylcholinesterase, (b) androgen receptor, (c) cyclooxygenase-2, (d) fibroblast growth factor receptor kinase, (e) enoyl ACP reductase, (f) tyrosine kinase SRC, (g) P38 mitogen activated protein, (h) platelet derived growth factor receptor kinase.

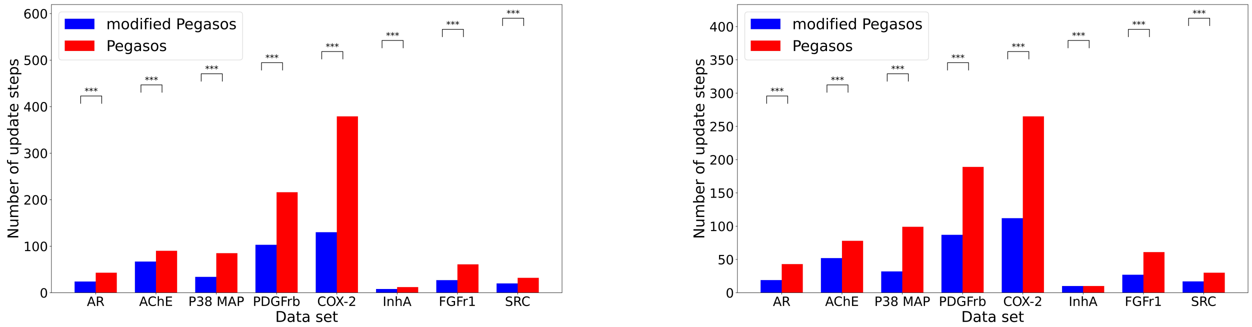

(a) Comparison between modified and standard Pegasos with the All Constraints Learning Strategy

(b) Comparison between modified and standard Pegasos with the Single Constraint Learning Strategy

Figure S3: Bar plot showing the mean number of update steps required to train the models to achieve an Adjusted Rand Index of 0.95, averaged over 1000 repetitions. The Morgan fingerprint with radius two is used as the descriptor. For each data set, the red bars represent the standard Pegasos algorithm and the blue bars represent the modified version. The number of update steps refers to the number of times the models were updated. Significance brackets indicate the significance of the differences of the number of learning steps of the modified and the standard Pegasos algorithm (paired sample t-test) with *** indicating an uncorrected p-value < 0.001.

5 Runtime results

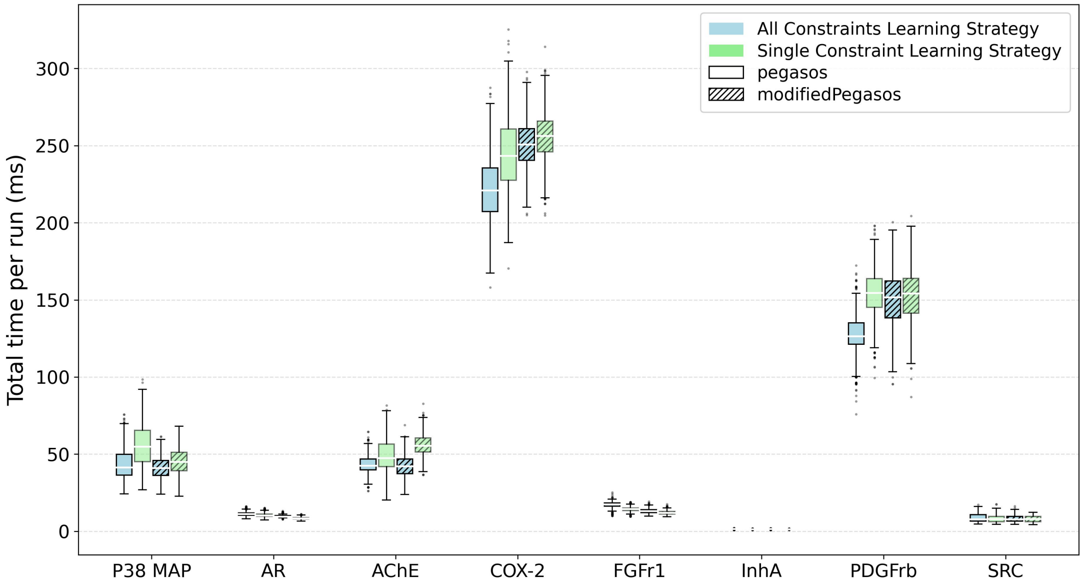

Figure S4: Distribution of runtime measurements in milliseconds for complete runs in milliseconds for the All Constraints Strategy (light blue) and Single Constraint Strategy (light green) and the methods Pegasos (solid) and modifiedPegasos (hatched) across the datasets for the targets P38 MAP, AR, AChE, COX-2 , FGFr1, InhA, PDGFrb, and SRC across 1,000 runs using the Morgan fingerprints descriptor with radius 4 and 1024 bits.

On average, running an experiment takes 64.64 milliseconds across all data sets. The maximum runtime is 325.26 milliseconds for the COX-2 data set when using the Pegasos method with the Single Constraint Learning Strategy, as shown in Figure S4.

6 Intra- and inter-cluster comparison

Figure S5 shows the mean intra-cluster distance versus the mean nearest-cluster distance for the remaining data sets.

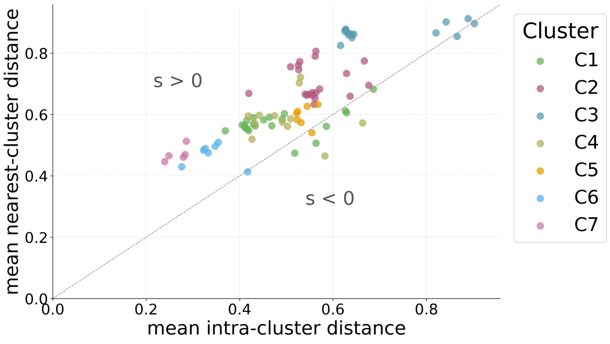

(a) AChE

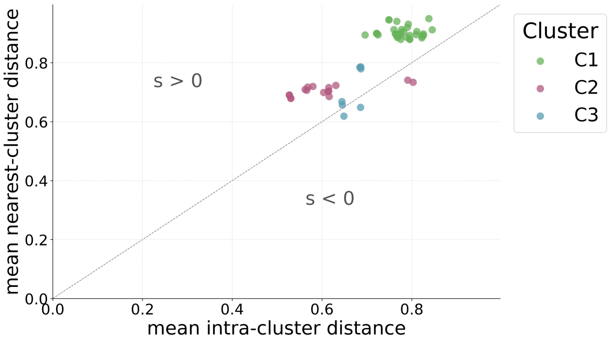

(b) AR

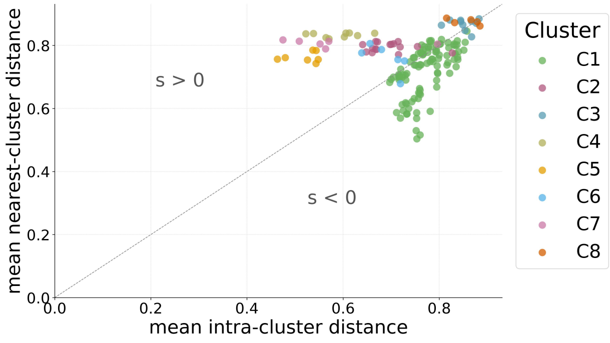

(c) COX-2

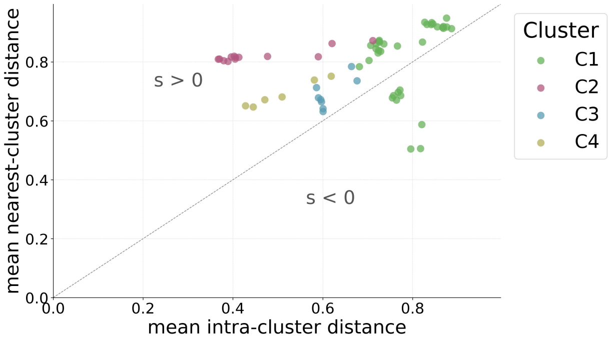

(d) FGFr1

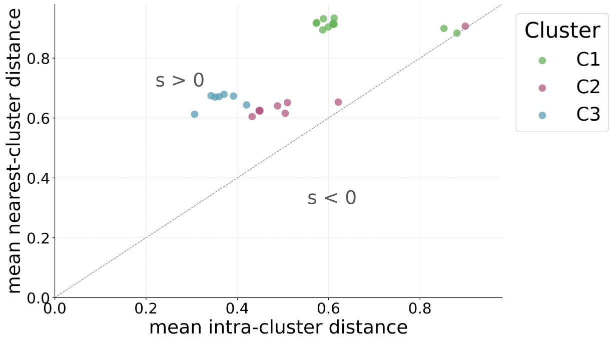

(e) InhA

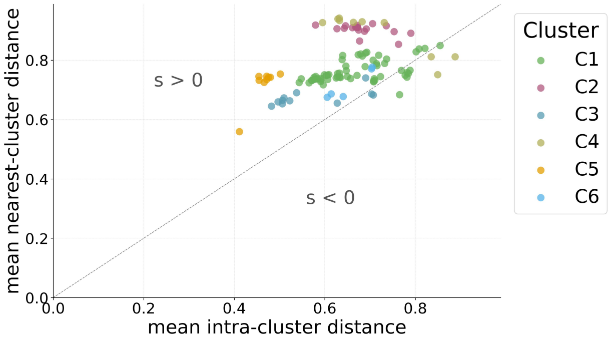

(f) P38 MAP

Figure S5: Scatter plot of mean intra-cluster distance versus mean nearest-cluster distance for all data sets. Each data point represents a molecule, colored by the cluster assignment of the expert clustering. Distances are computed as 1 - Tanimoto similarity[S1] using Morgan fingerprints with radius 4 and 1024 bits. A silhouette of $s < 0$ indicates that molecules are closer to a neighboring cluster than to their own. Results are shown for the targets (a) acetylcholinesterase, (b) androgen receptor, (c) cyclooxygenase-2, (d) fibroblast growth factor receptor kinase, (e) enoyl ACP reductase, (f) P38 mitogen activated protein

7 Adjusted Rand Index convergence behavior

Figure S6 displays the Adjusted Rand Index convergence behavior with the modified Pegasos method and the All Constraints Learning Strategy. Figure S7 displays the Adjusted Rand Index convergence behavior with the Pegasos method and the All Constraints Learning Strategy. Figure S8 displays the Adjusted Rand Index convergence behavior with the modified Pegasos method and the Single Constraint Learning Strategy. Figure S9 displays the Adjusted Rand Index convergence behavior with the Pegasos method and the Single Constraint Learning Strategy.

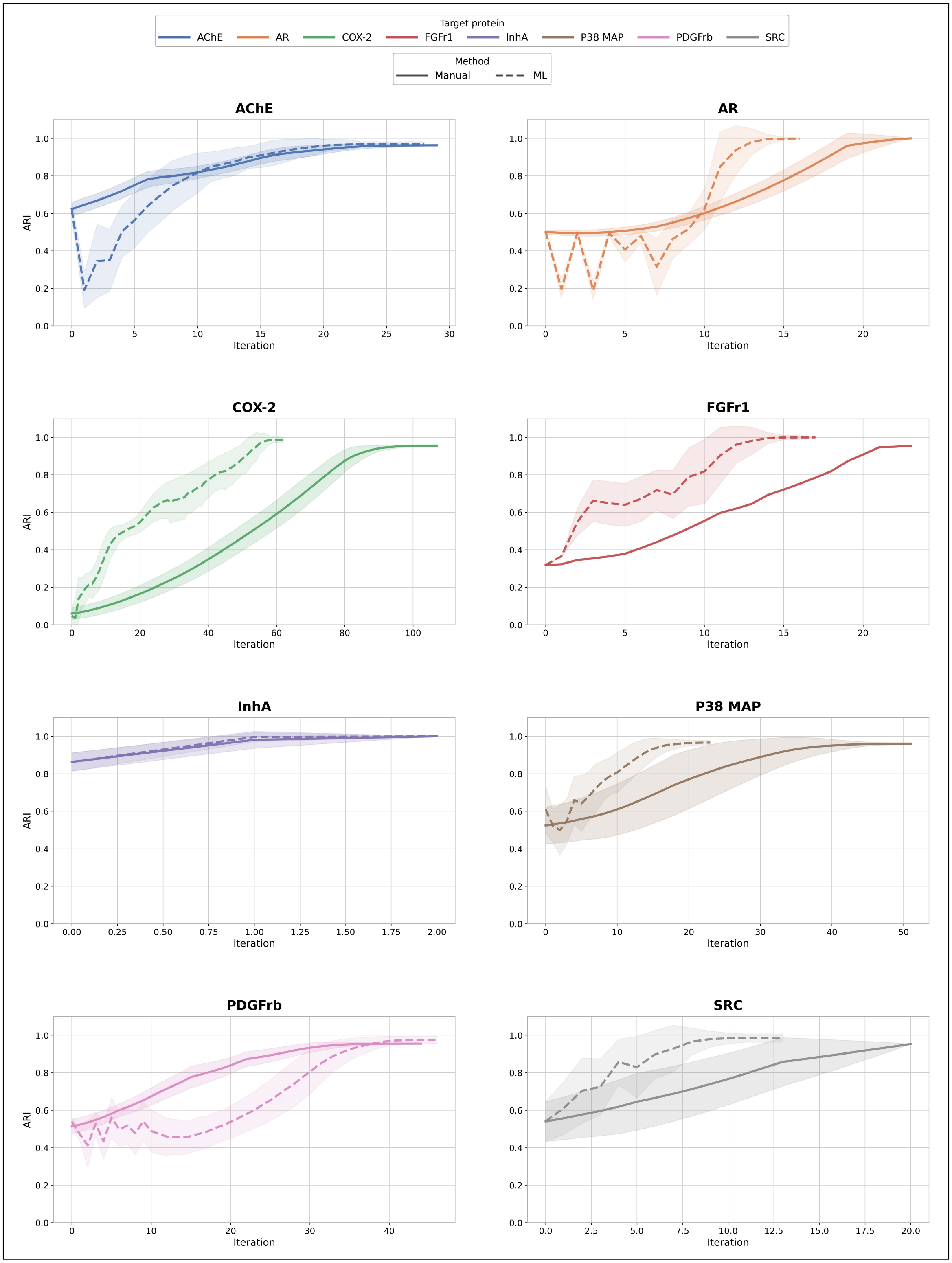

Figure S6: The progression of the Adjusted Rand Index for the experiments using Morgan Fingerprints with a radius of four. Results are shown for the All Constraints Learning Strategy and modified Pegasos. The bold lines represent the mean Adjusted Rand Index, and the shaded areas indicate the standard deviation. The solid lines represent the manual approach and the dashed lines the machine learning approach.

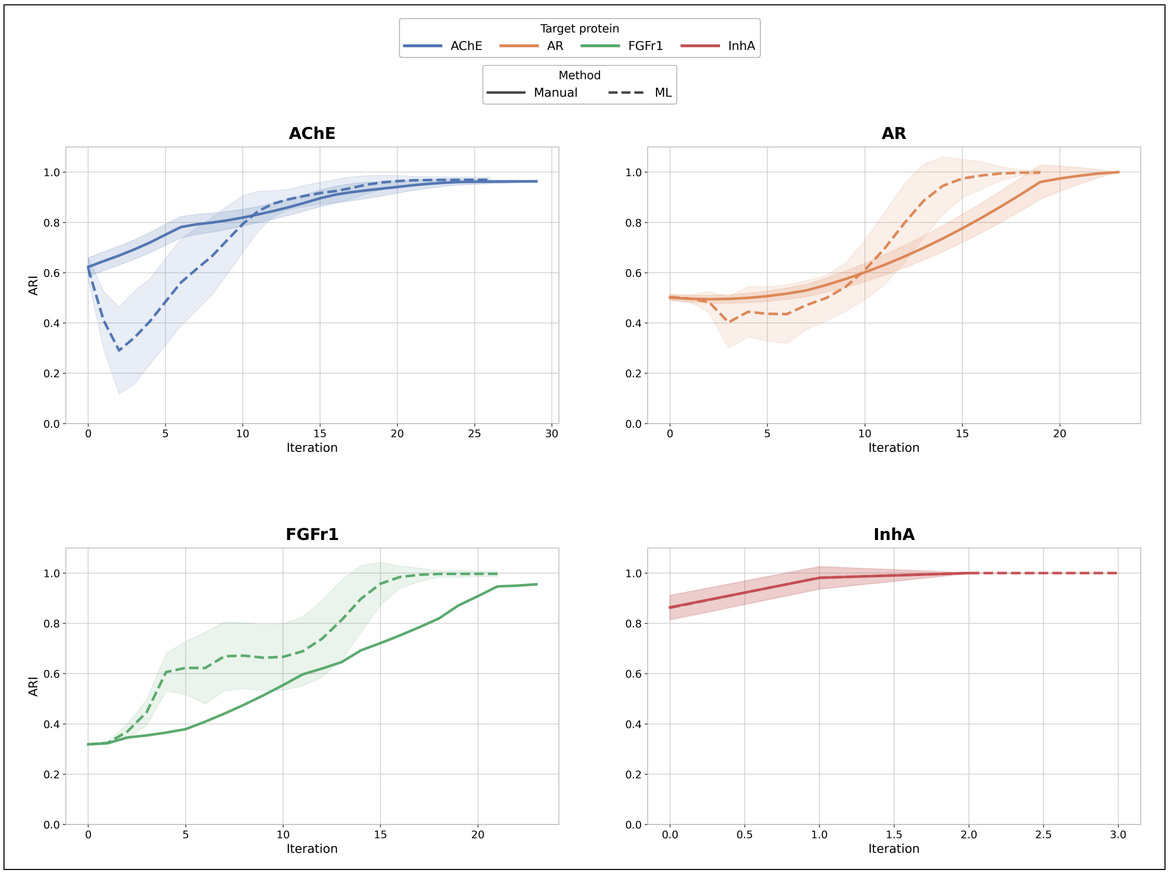

Figure S7: The progression of the Adjusted Rand Index for the experiments using Morgan Fingerprints with a radius of four. Results are shown for the All Constraints Learning Strategy and Pegasos and the targets acetylcholinesterase, androgen receptor, enoyl ACP reductase, fibroblast growth factor receptor kinase. The bold lines represent the mean Adjusted Rand Index, and the shaded areas indicate the standard deviation. The solid lines represent the manual approach and the dashed lines the machine learning approach.

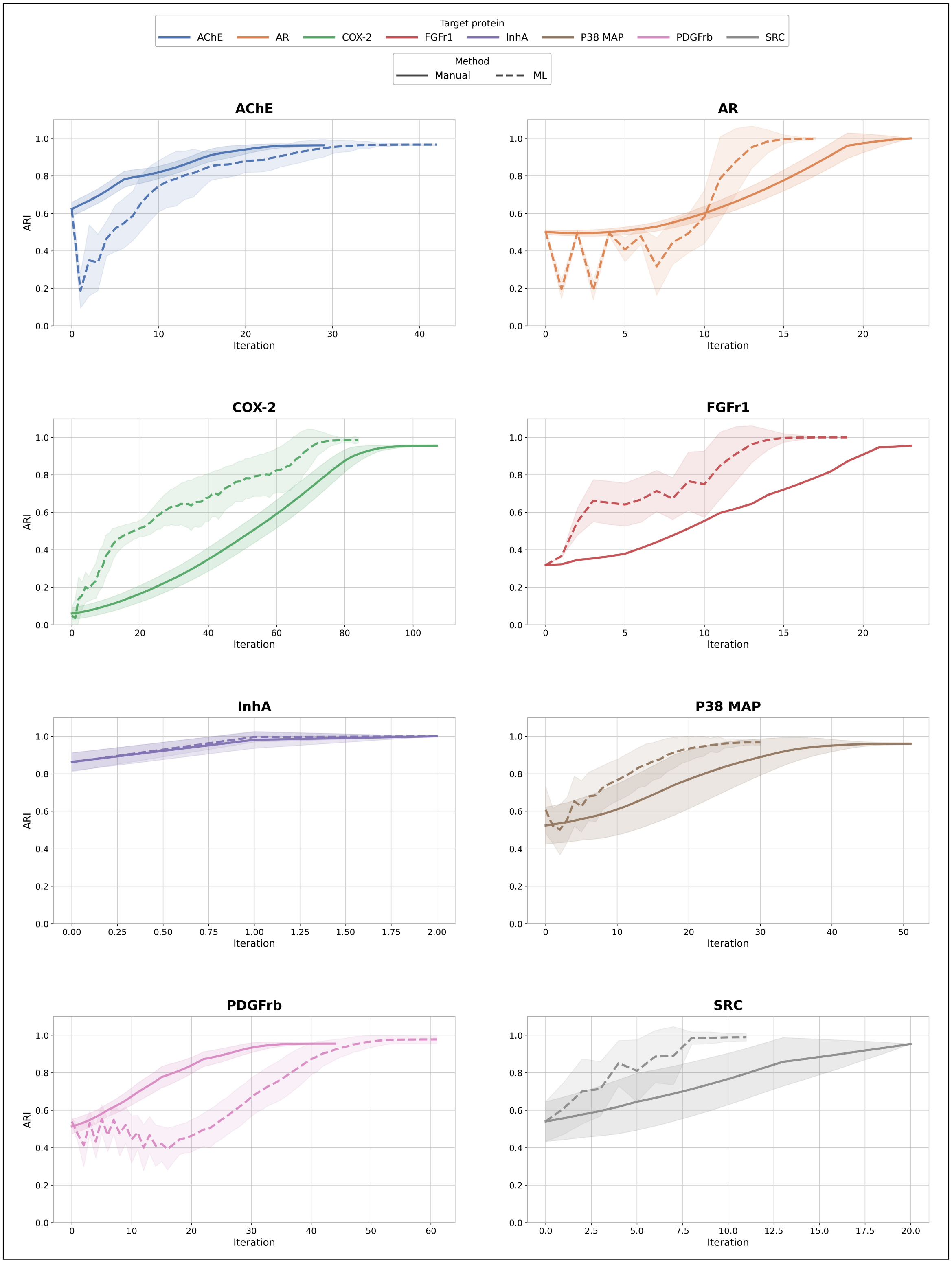

Figure S8: The progression of the Adjusted Rand Index for the experiments using Morgan Fingerprints with a radius of four. Results are shown for the Single Constraint Learning Strategy and modified Pegasos. The bold lines represent the mean Adjusted Rand Index, and the shaded areas indicate the standard deviation. The solid lines represent the manual approach and the dashed lines the machine learning approach.

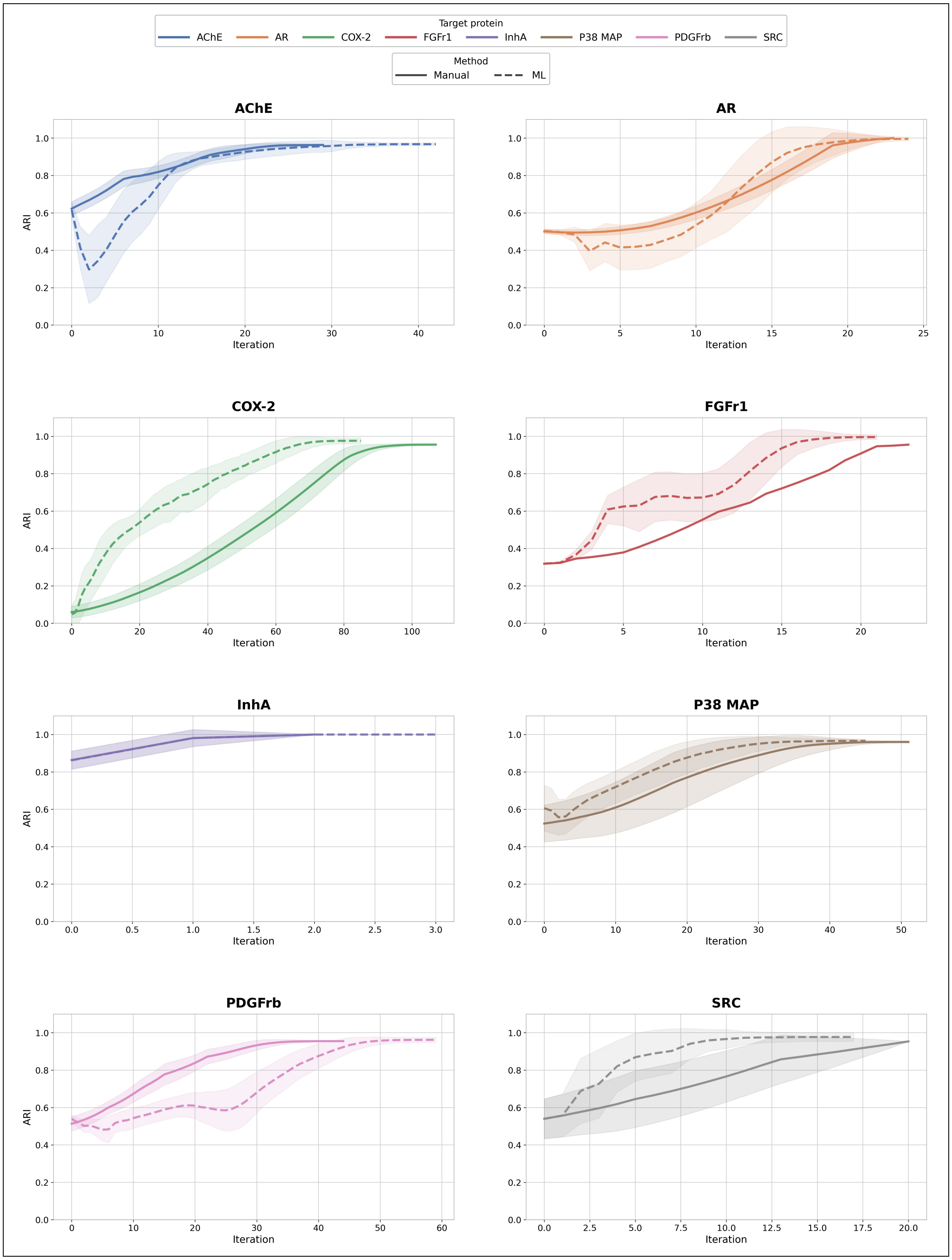

Figure S9: The progression of the Adjusted Rand Index for the experiments using Morgan Fingerprints with a radius of four. Results are shown for the Single Constraint Learning Strategy and Pegasos . The bold lines represent the mean Adjusted Rand Index, and the shaded areas indicate the standard deviation. The solid lines represent the manual approach and the dashed lines the machine learning approach.

8 Web server experiment SRC data set

Figure S10 shows a use case that illustrates how PegaPlus can support and accelerate expert clustering on the example of the tyrosine kinase SRC target from the DUD database.[S2] The clustering by Andrew Good et al., based on reduced graphs and manual inspection,[S3] was used as the ground truth clustering. Constraints are displayed with a thick black border. The following parameters were used for this experiment: For the SPE, a learning rate of 0.5, 2,000 iterations, and 5,000 cycles were used. The Online SVM was used with a learning rate of 2.0 and combined learning with embedding. The Morgan fingerprint with a radius of 4 was used as a descriptor.

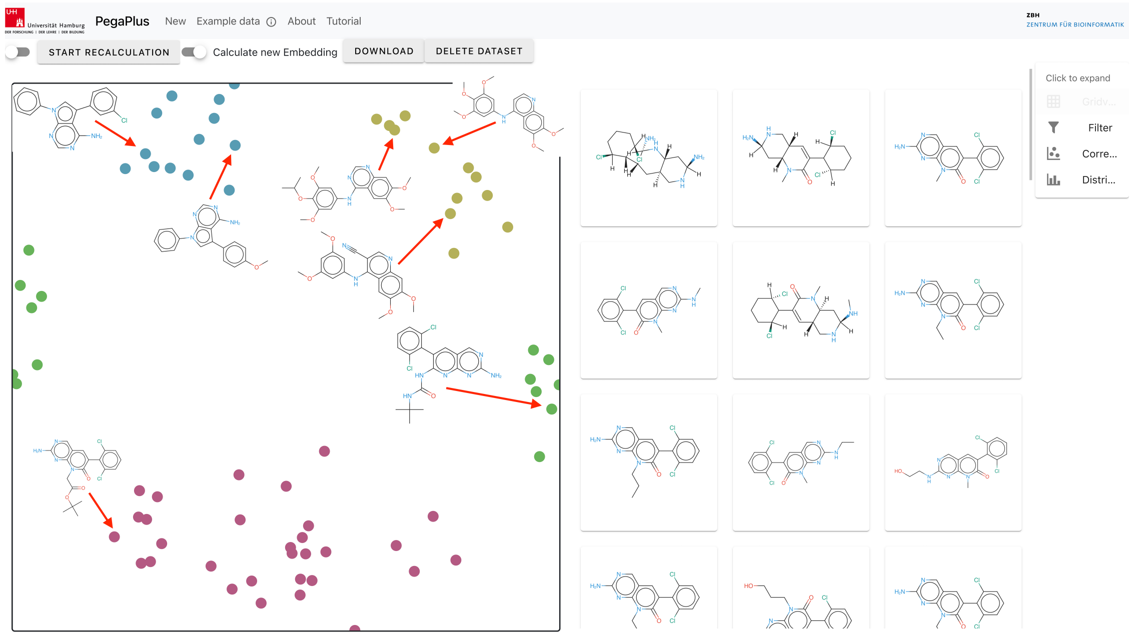

(a) The first molecule requiring reassignment to a different cluster is depicted in the lower left corner and represented as a magenta colored circle.

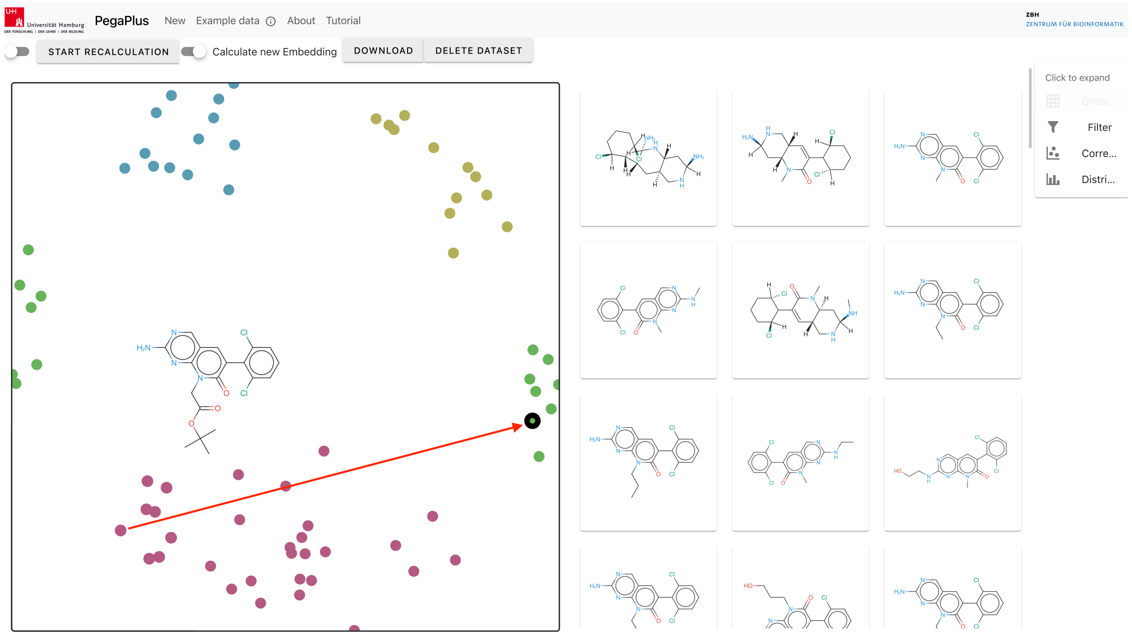

(b) The molecule was moved to the green cluster, and a new calculation was started.

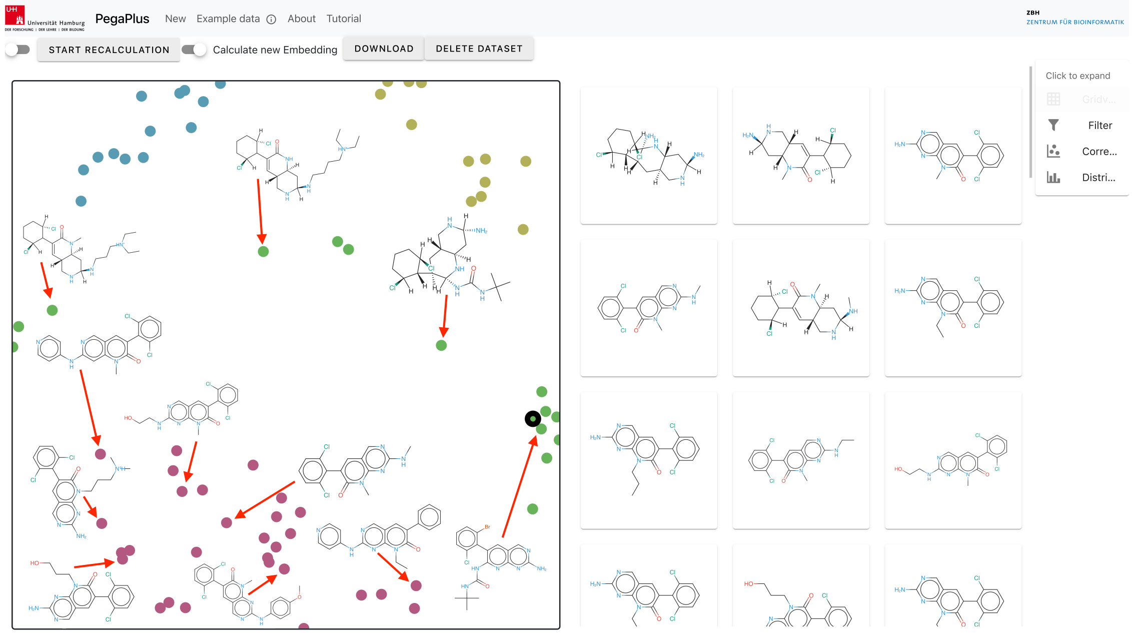

(c) The new results were examined, revealing that a molecule in the magenta cluster should be moved to the green cluster.

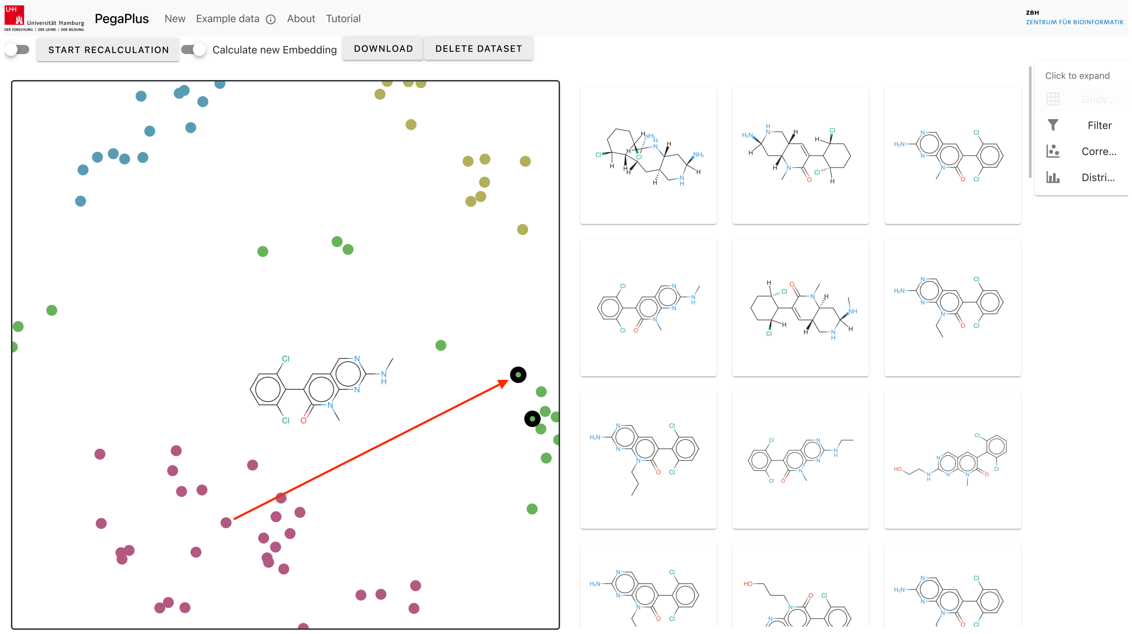

(d) The molecule was moved to the green cluster and a new calculation was started.

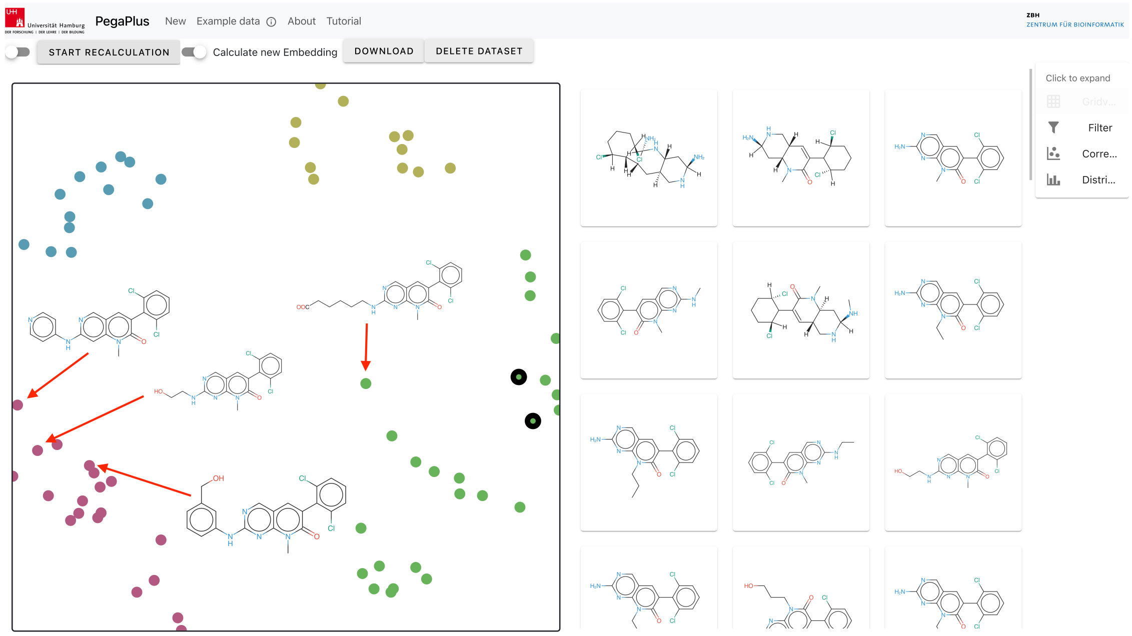

(e) The new results were examined, revealing that another molecule in the magenta cluster should be moved to the green cluster.

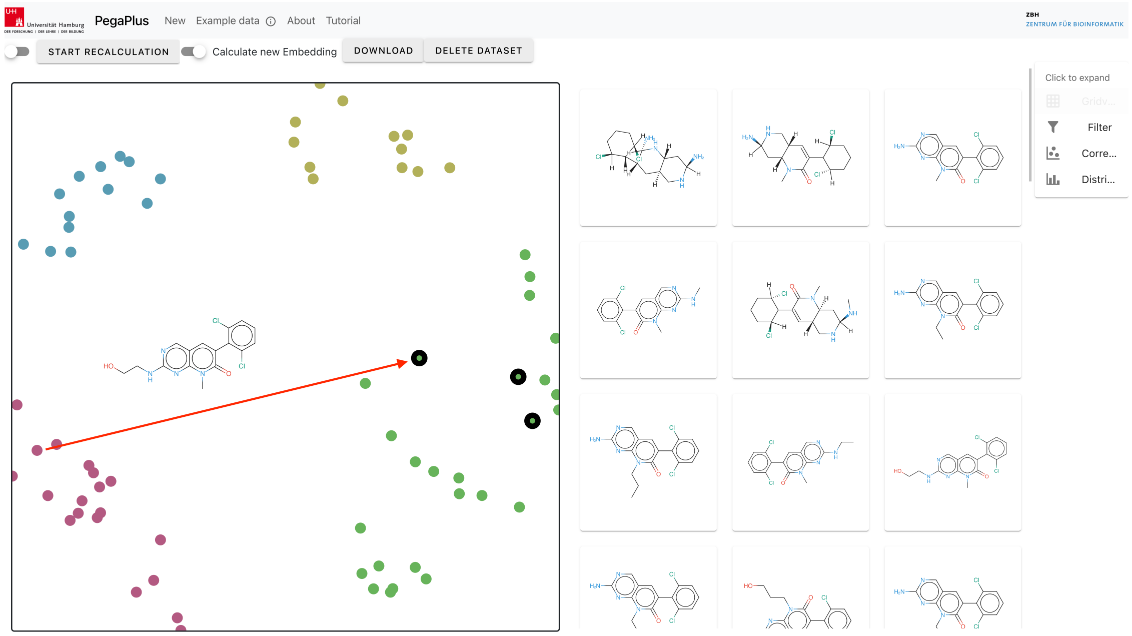

(f) The molecule was moved to the green cluster, and a new calculation was started.

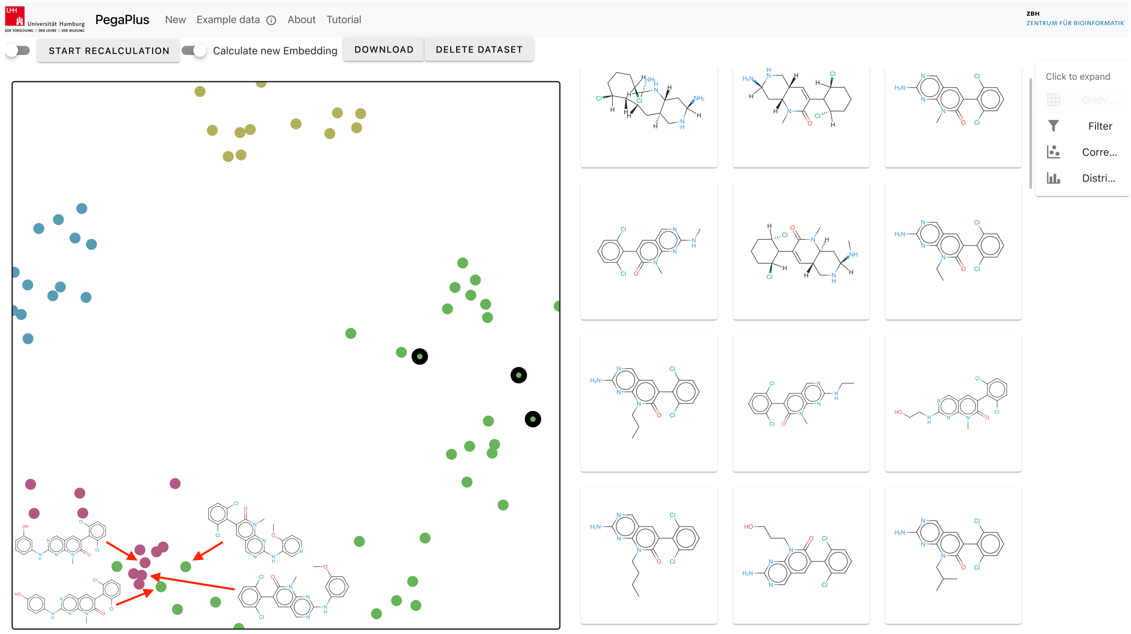

(g) Two clusters overlapped at one point in the new result. This point was examined, and a molecule was identified that belongs to the magenta cluster rather than the green one.

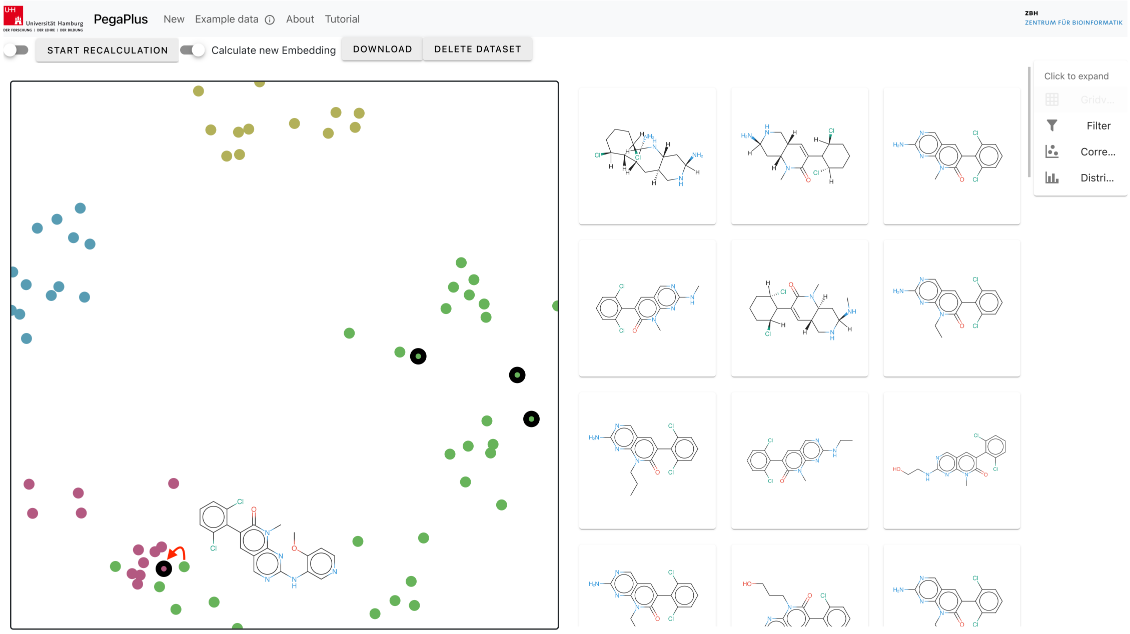

(h) The molecule was moved to the magenta cluster, and a new calculation was started.

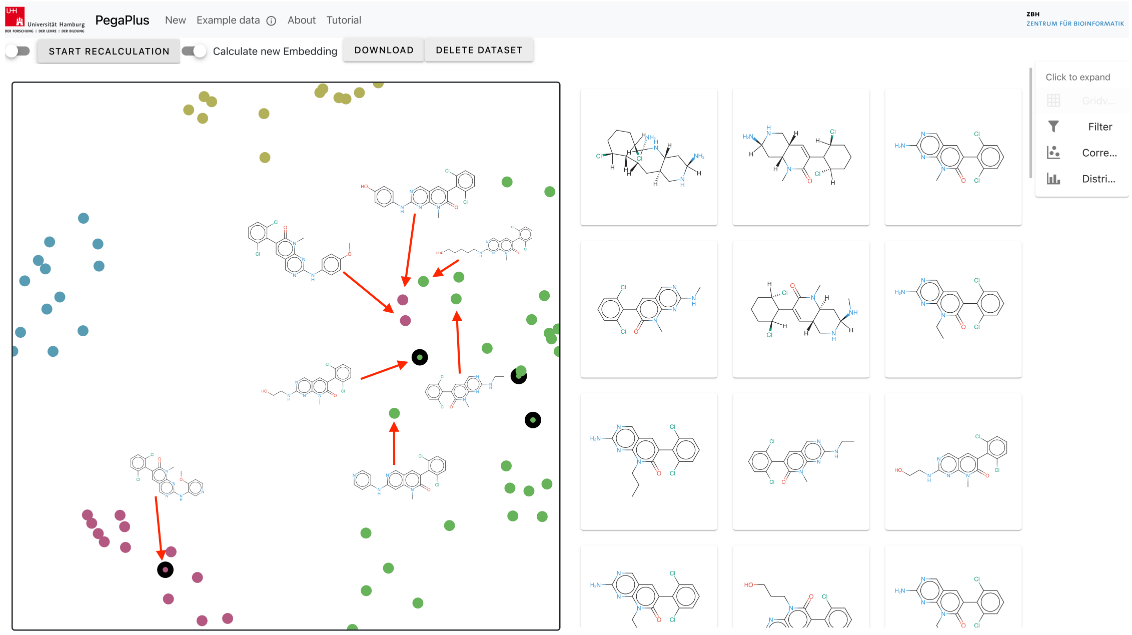

(i) Once again, there was an overlap between two clusters. The two magenta molecules near the green cluster are assigned to the correct cluster but were embedded near the wrong cluster. The proximity was further investigated, and a molecule was identified that had been incorrectly clustered.

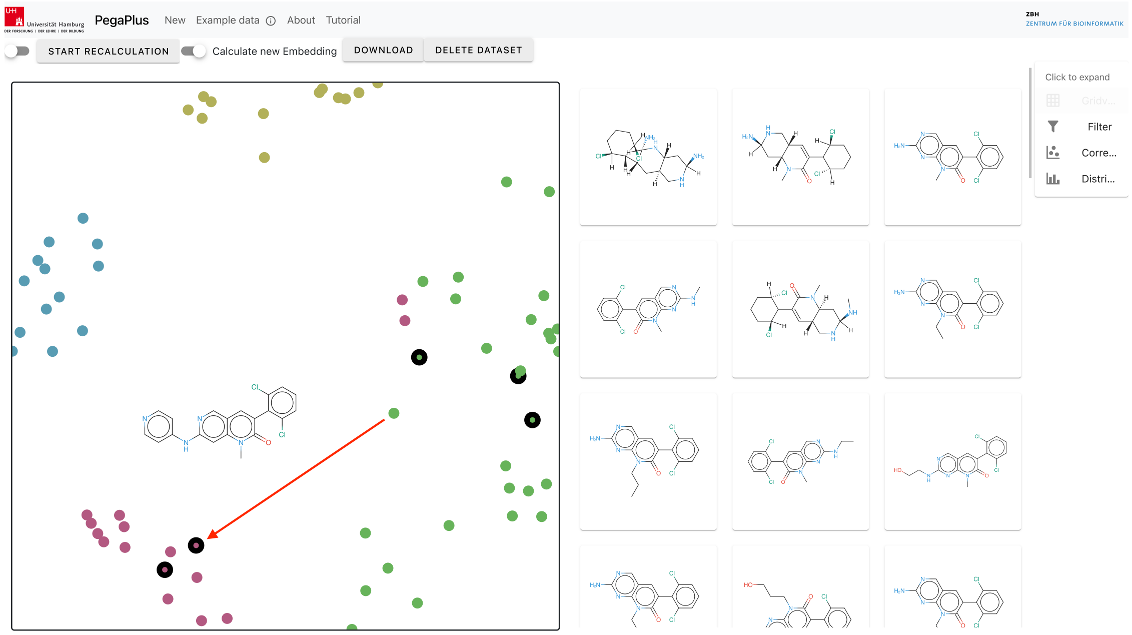

(j) The molecule was moved to the magenta cluster and a new calculation was started.

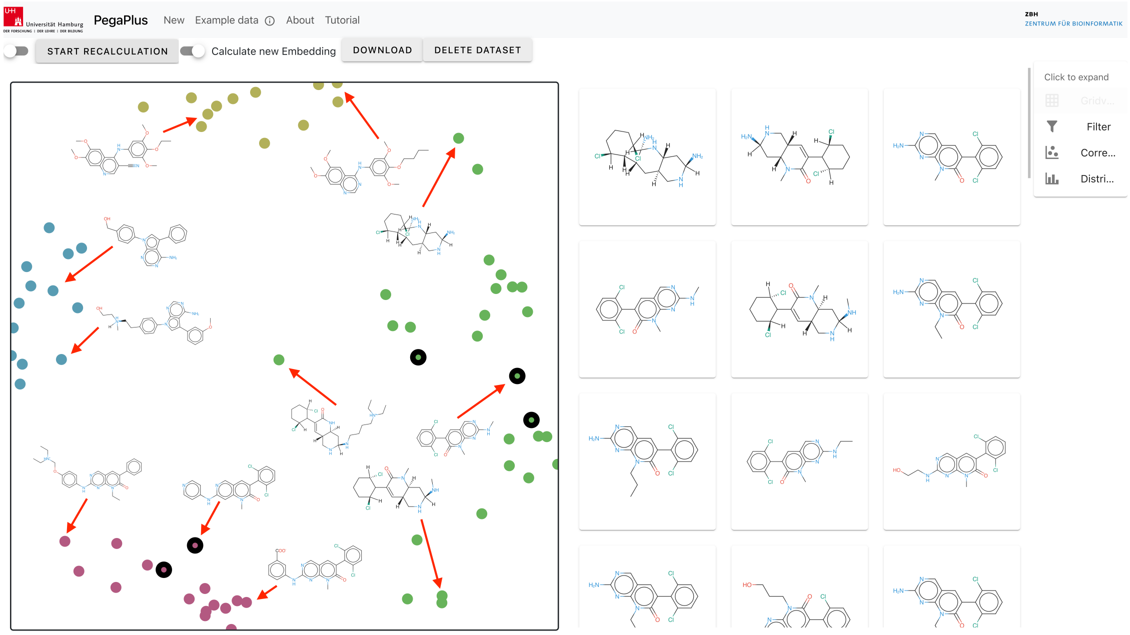

(k) The final result in which every molecule was correctly clustered following expert clustering.

Figure S10: Figures (a) through (k) illustrate a clustering process aimed at achieving the expert clustering. In this scenario, five steps were required for the SRC dataset. Note that intermediate steps in the clustering process, such as filtering molecules, are not shown. However, this does not mean that these steps were not performed. Molecules are displayed as colored circles on the drawing plane, with their respective cluster colors. Constraints are displayed with thick black borders. The red arrow in (b), for example, indicates motion. In (a), however, it merely indicates the position of the corresponding molecular structure. A circle at the start of an arrow, for instance, in (b) indicates the molecule's initial position, while the circle at the end shows its new position before recalculation.

9 Web server example adding clusters

Figure S11 illustrates how PegaPlus can handle an incorrect number of clusters compared to an expert clustering, i.e. when starting with two initial clusters instead of four. In this scenario PegaPlus must split up specific data points from the existing clusters and create new clusters. The example used the tyrosine kinase SRC target from the DUD database.[S2] The clustering by Andrew Good et al., based on reduced graphs and manual inspection,[S3] was utilized as the ground truth clustering. Constraints are displayed with a thick black border. The following parameters were used for this experiment: For the SPE, a learning rate of 0.5, 2,000 iterations, and 5,000 cycles were used. The Online SVM was used with a learning rate of 2.0 and combined learning with embedding. The Morgan fingerprint with a radius of 4 was used as a descriptor. A total of 17 constraints were necessary to achieve the target clustering.

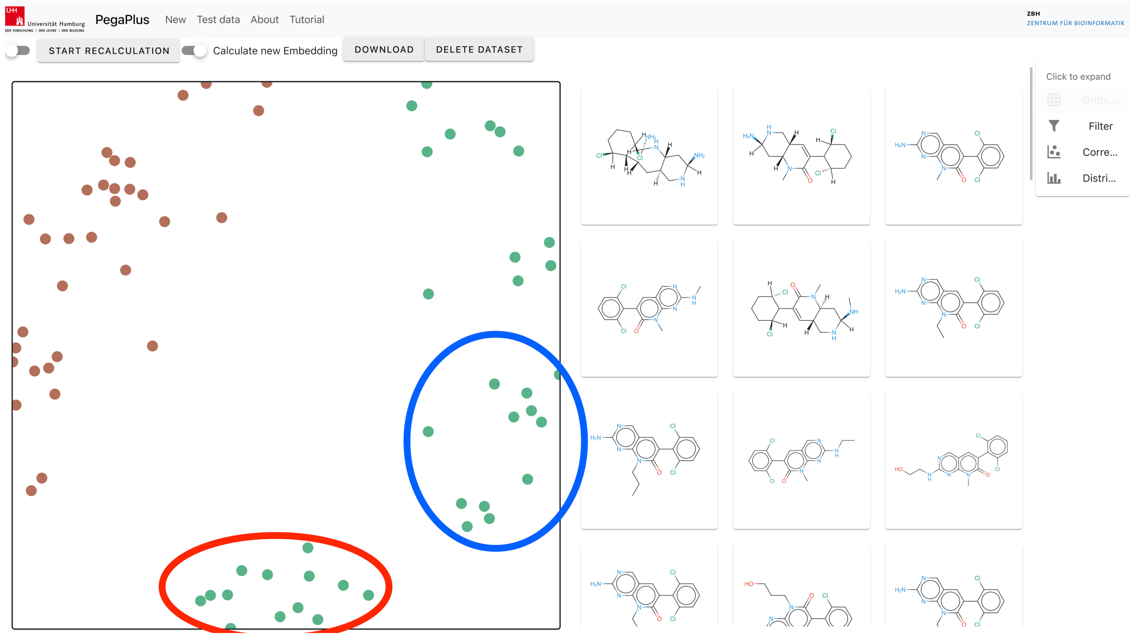

(a) This is the result of the first calculation using K-means++ and online SVM. The blue and red ellipses indicate clusters three and four, which are hidden inside the green cluster.

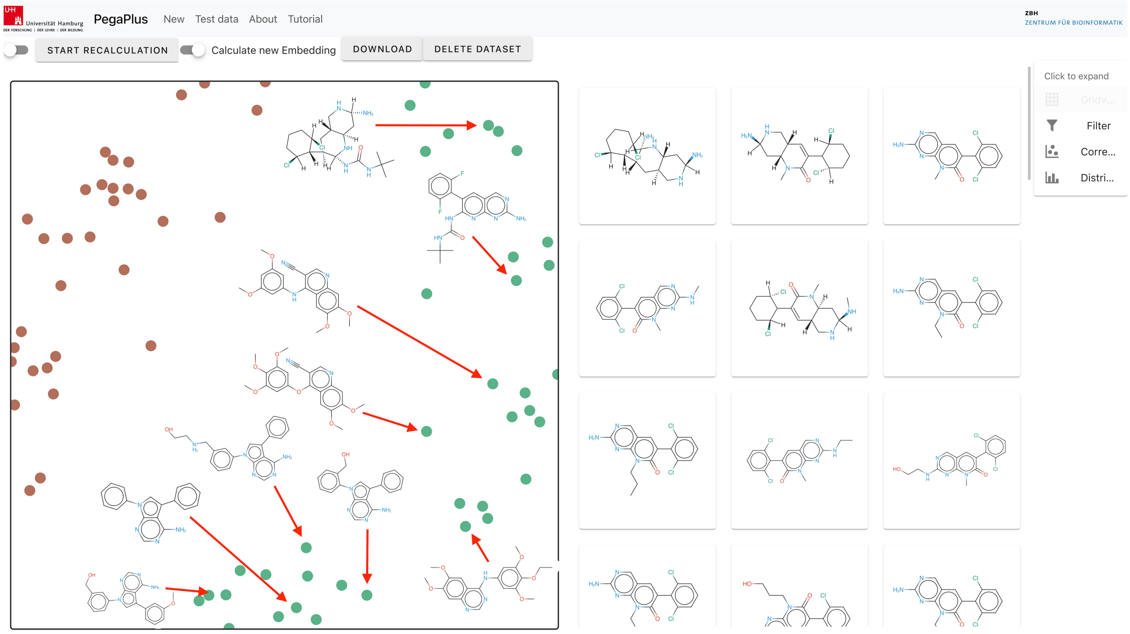

(b) An overview of the molecules of the green cluster.

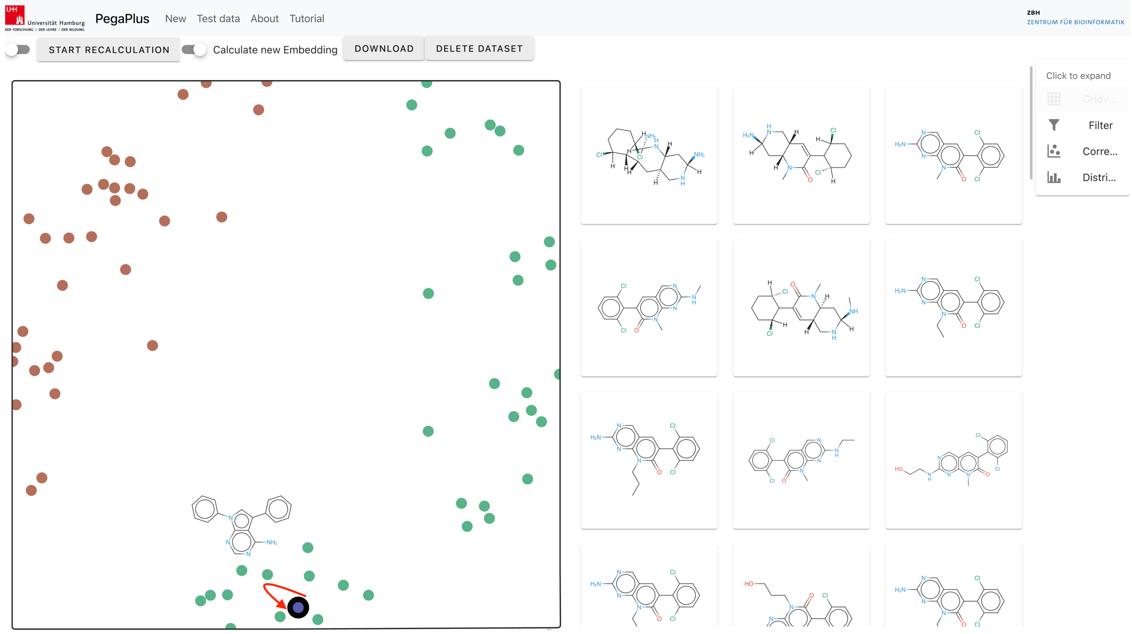

(c) The first new created cluster.

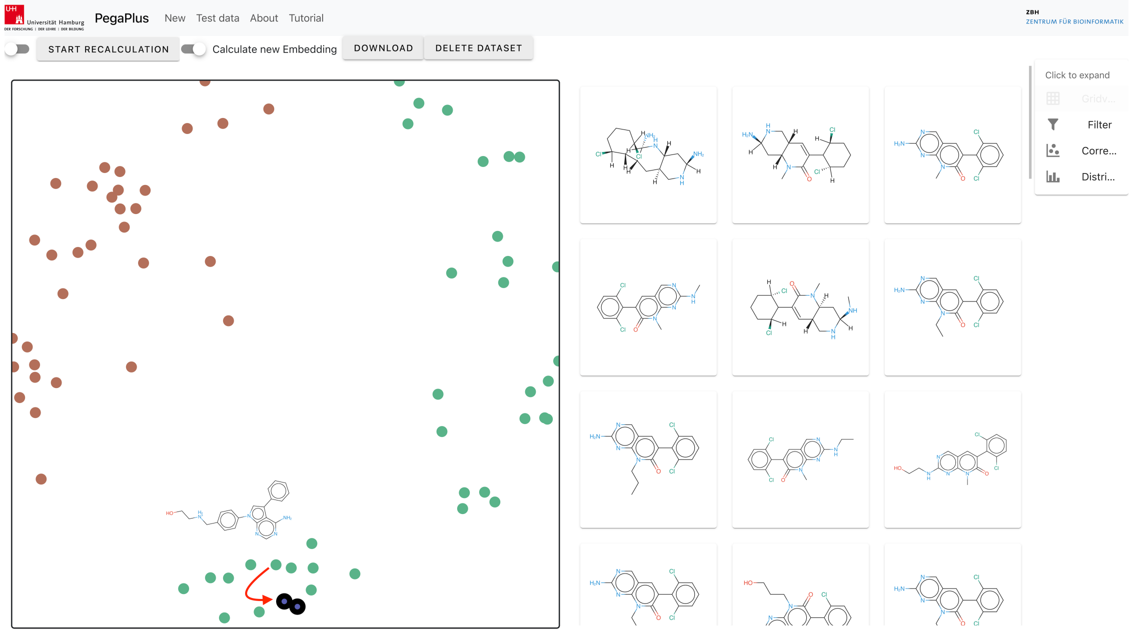

(d) This is the clustering result after recalculation. Then, a second molecule is assigned to the purple cluster.

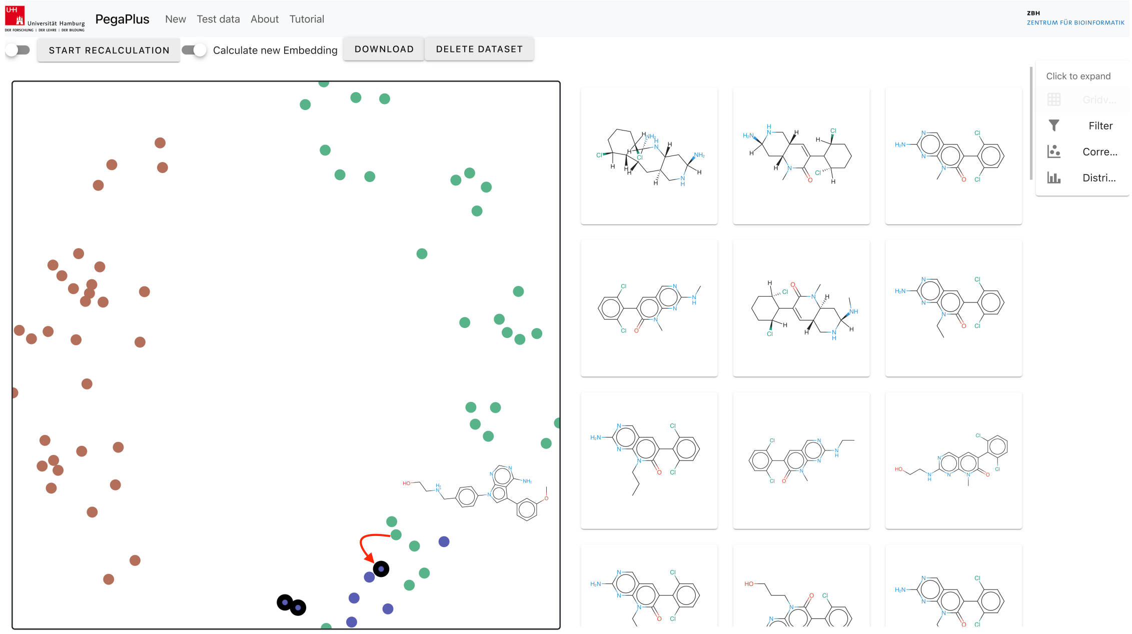

(e) After another calculation, a different molecule is assigned to the purple cluster.

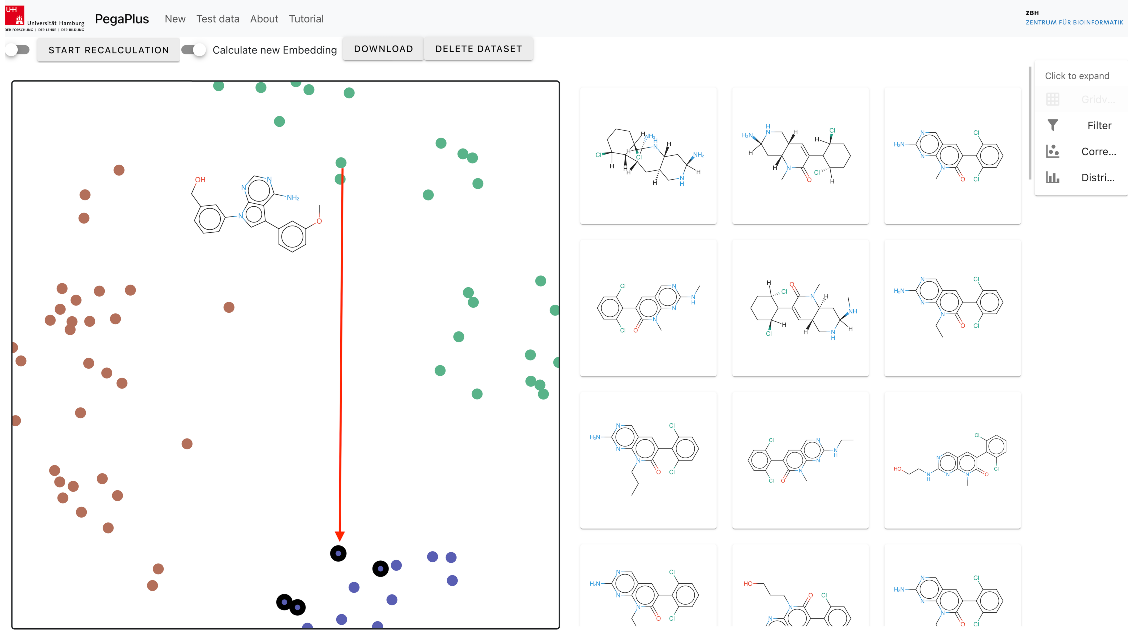

(f) A new clustering is shown, which was constructed through a recalculation. Another molecule has been assigned to the purple cluster.

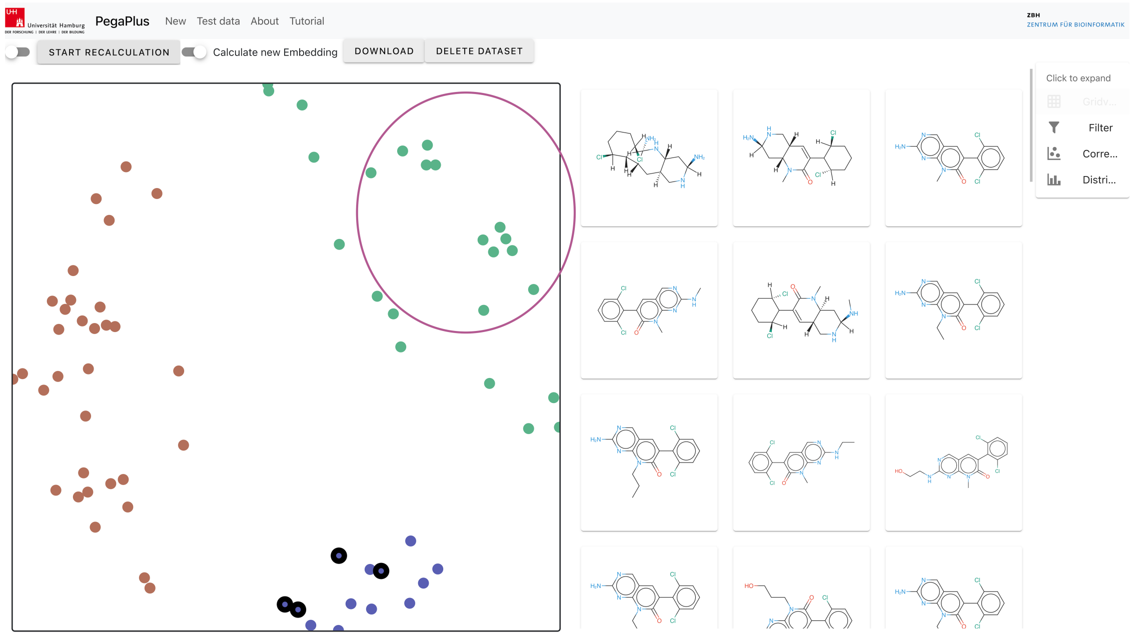

(g) After another recalculation, the purple cluster is fully constructed with four constraints. The magenta circle indicates the location of the fourth hidden cluster.

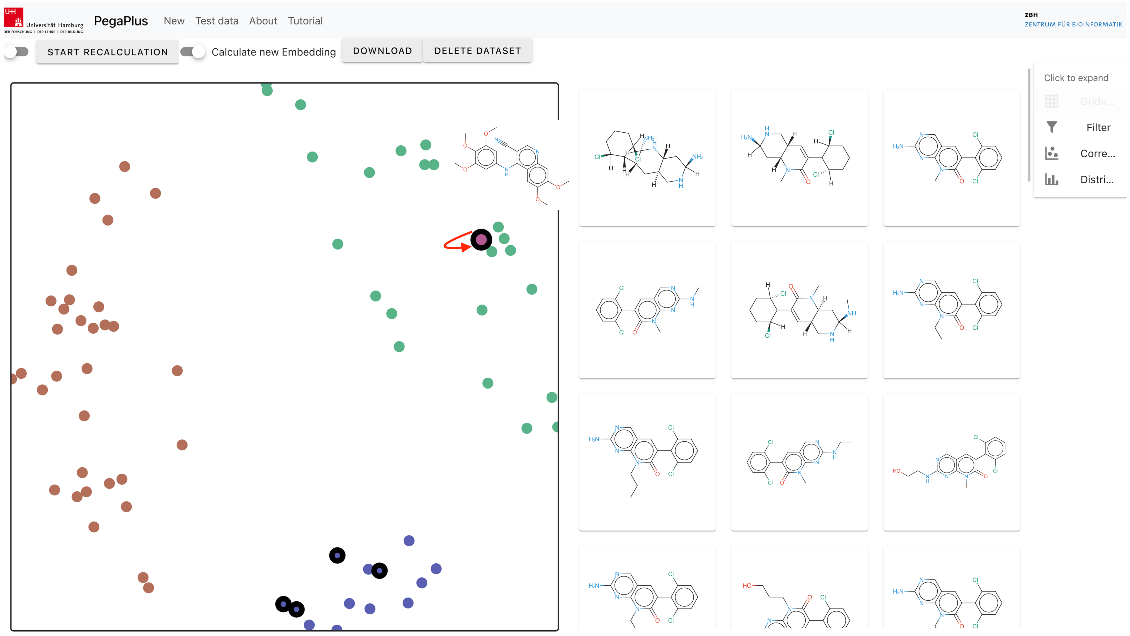

(h) The magenta cluster is created using a molecule in the center of the hidden cluster.

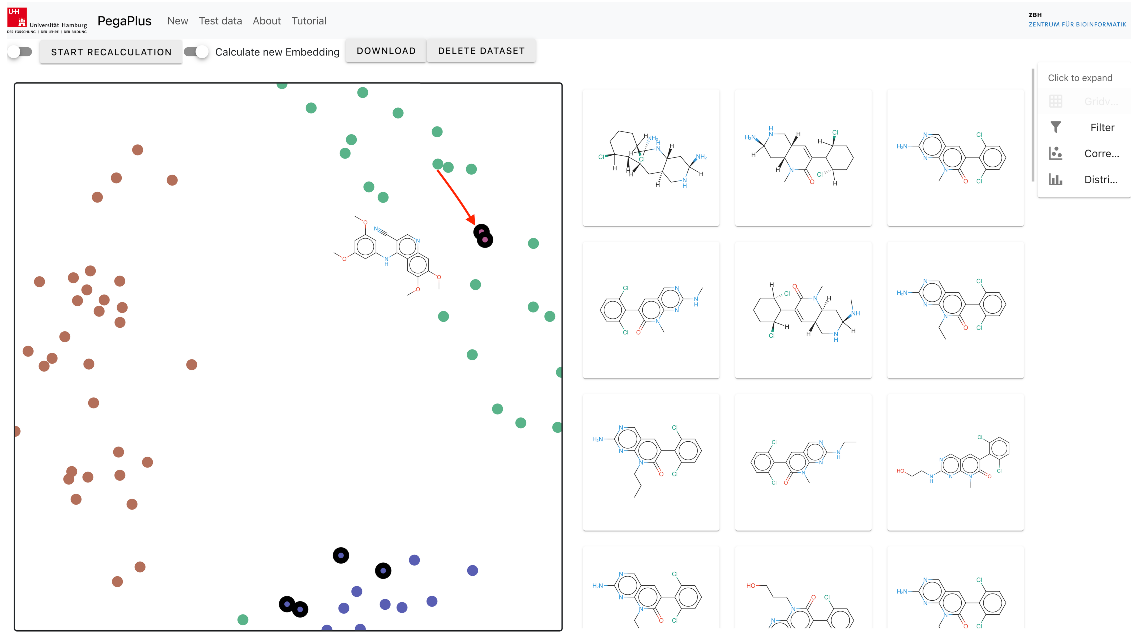

(i) After recalculation, a second molecule is assigned to the magenta cluster.

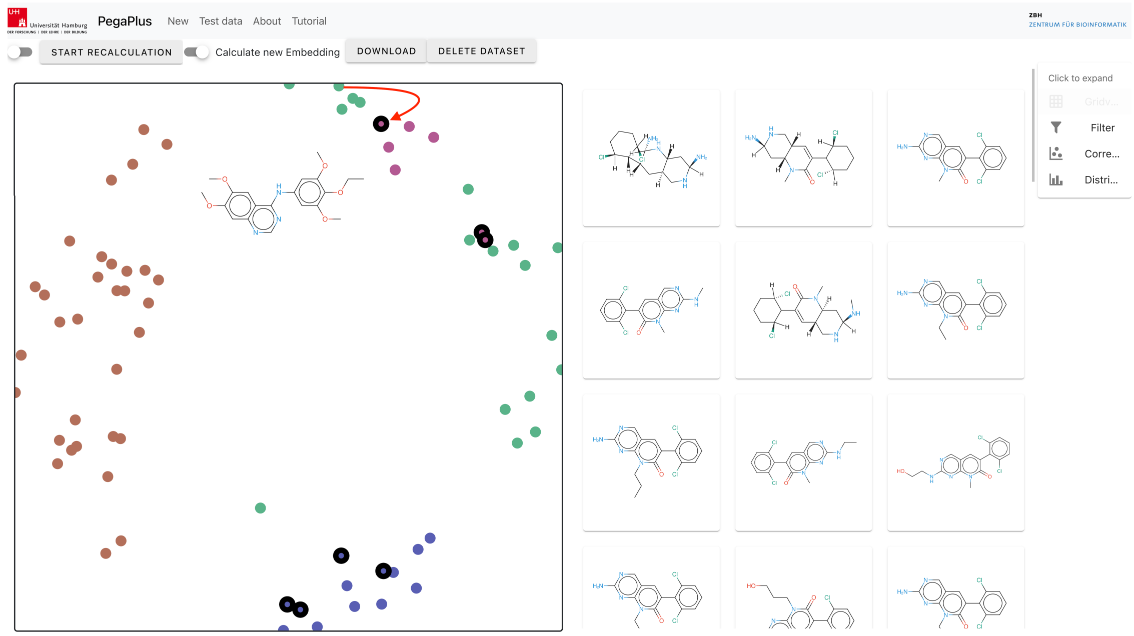

(j) This is the clustering result after another recalculation. Then, a third molecule has been assigned to the magenta cluster.

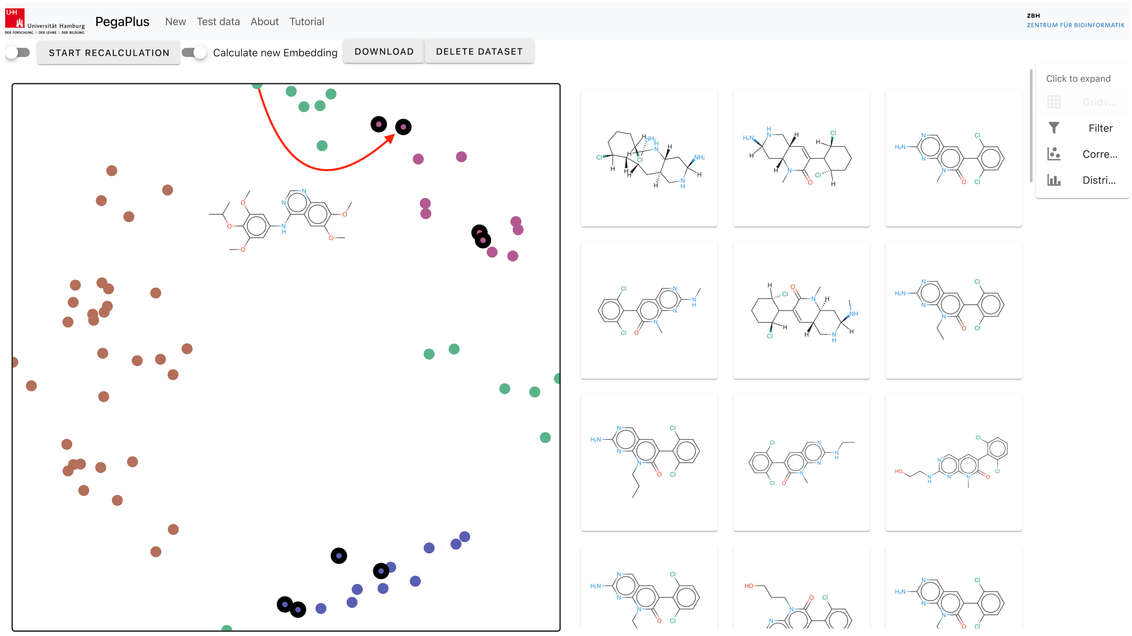

(k) A new clustering result is shown after recalculation, and a molecule is assigned to the magenta cluster.

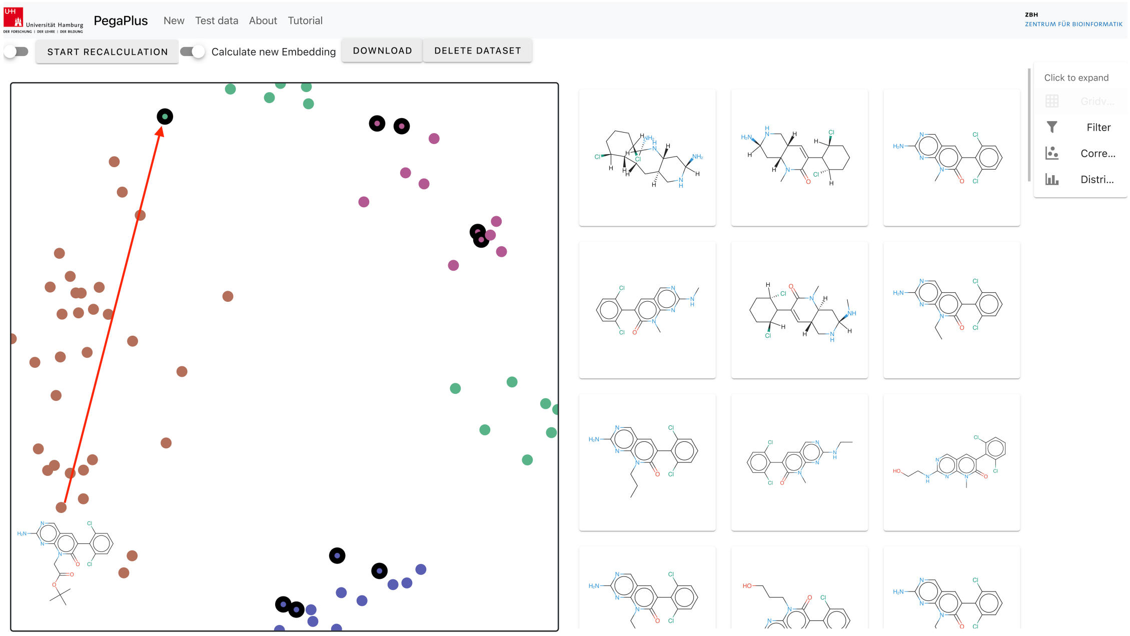

(l) The magenta cluster is fully created with four constraints and another recalculation. Then, a molecule is assigned from the brown cluster to the green one.

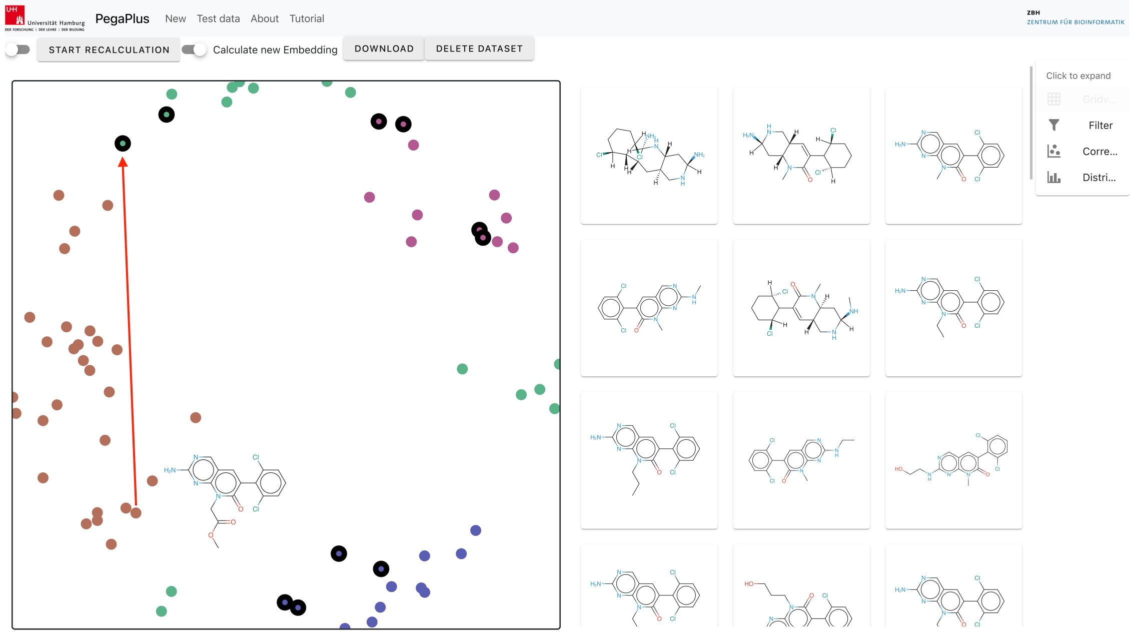

(m) After another recalculation, a different molecule is assigned to the green cluster.

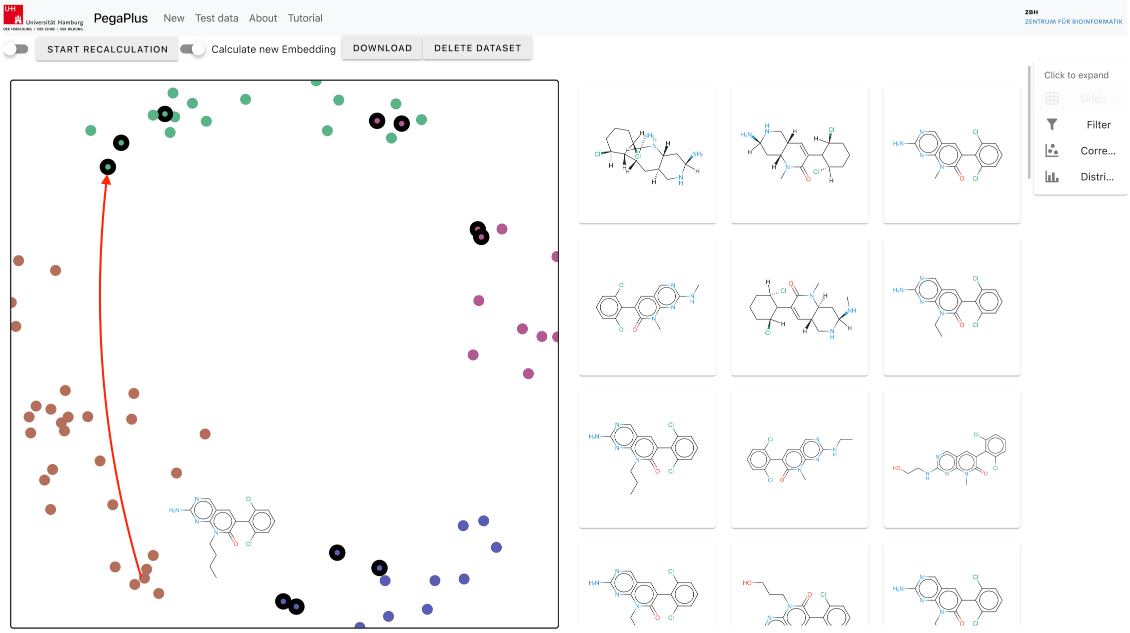

(n) The clustering result is shown after the recalculation. Another molecule has been assigned to the green cluster.

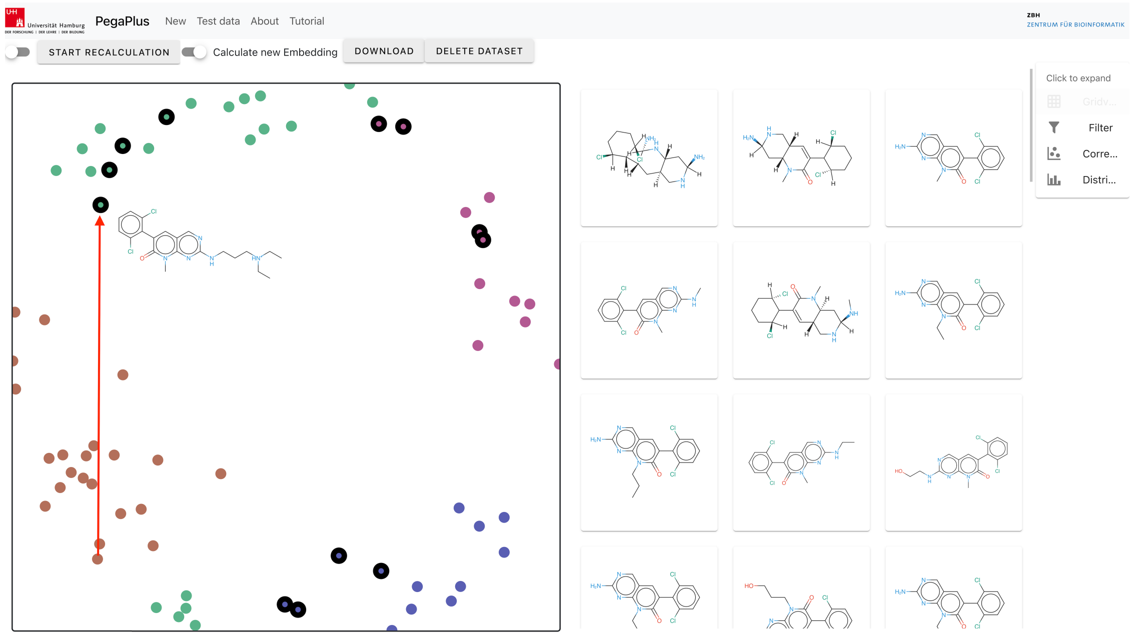

(o) A new clustering is shown, which was constructed through a recalculation. Another molecule has been assigned to the green cluster.

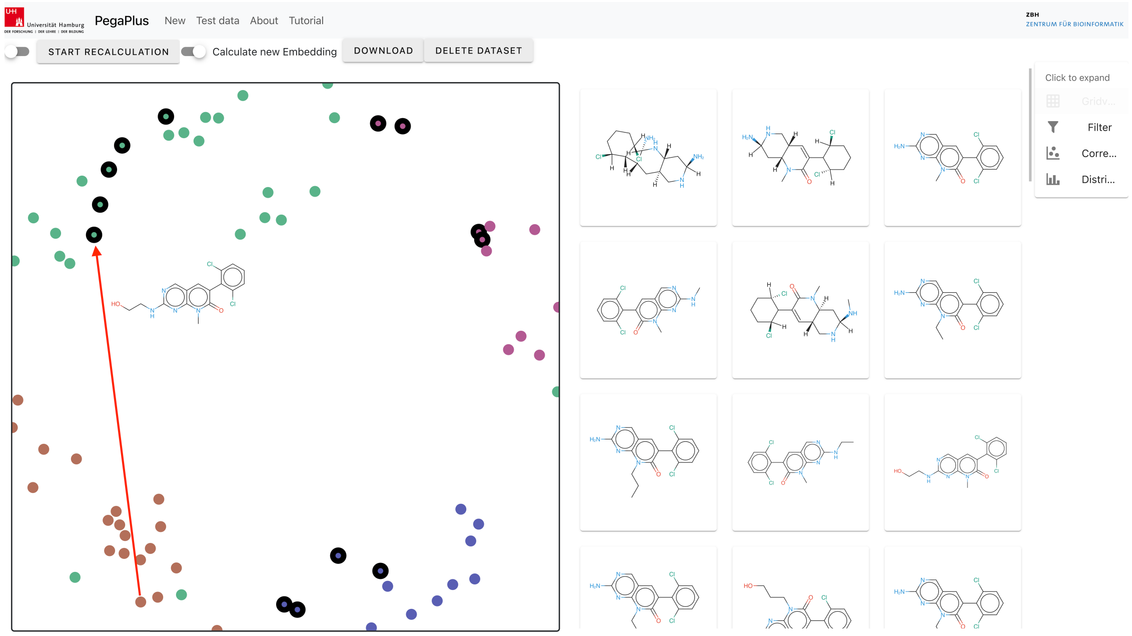

(p) After another recalculation, a different molecule is assigned to the green cluster.

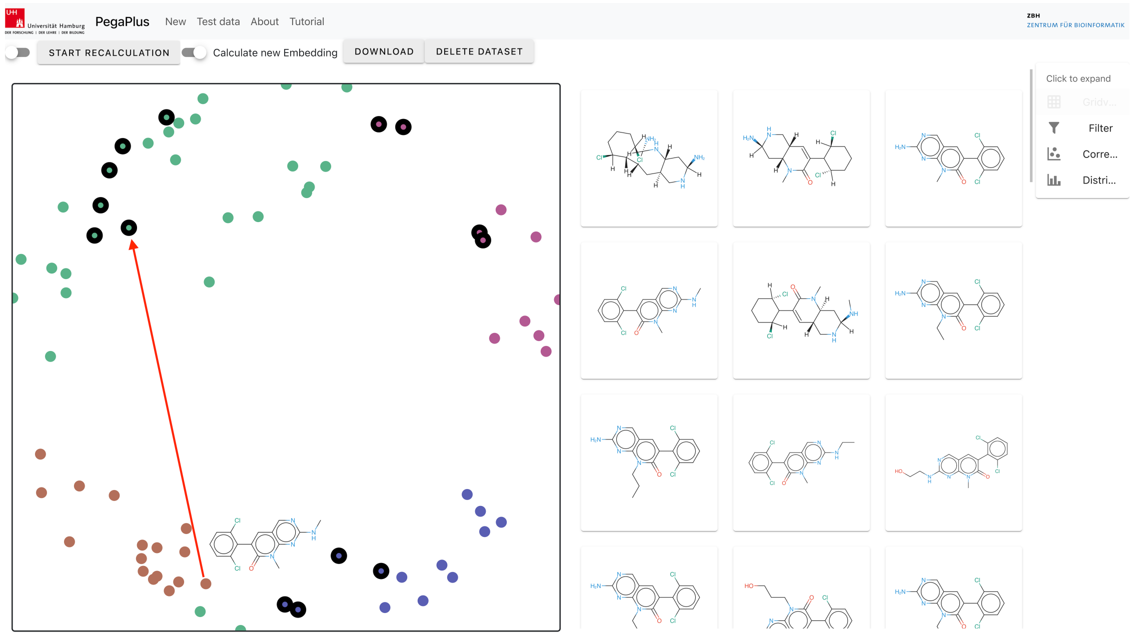

(q) This is the clustering result after another recalculation. Then, a third molecule has been assigned to the green cluster.

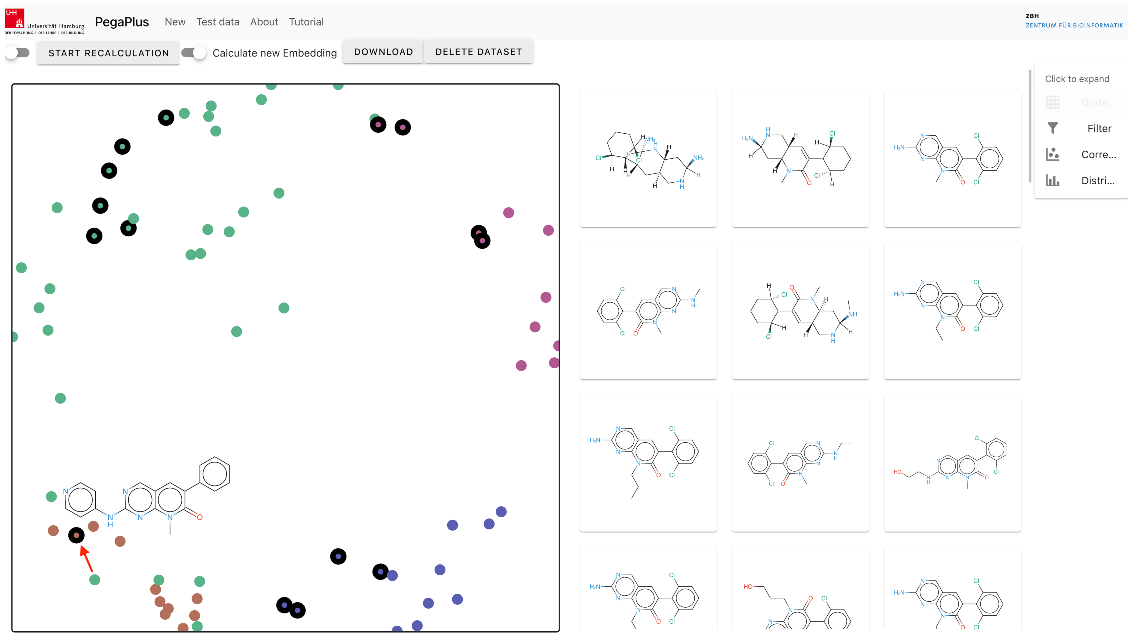

(r) After another calculation, a molecule is reassigned from the green cluster to the brown cluster.

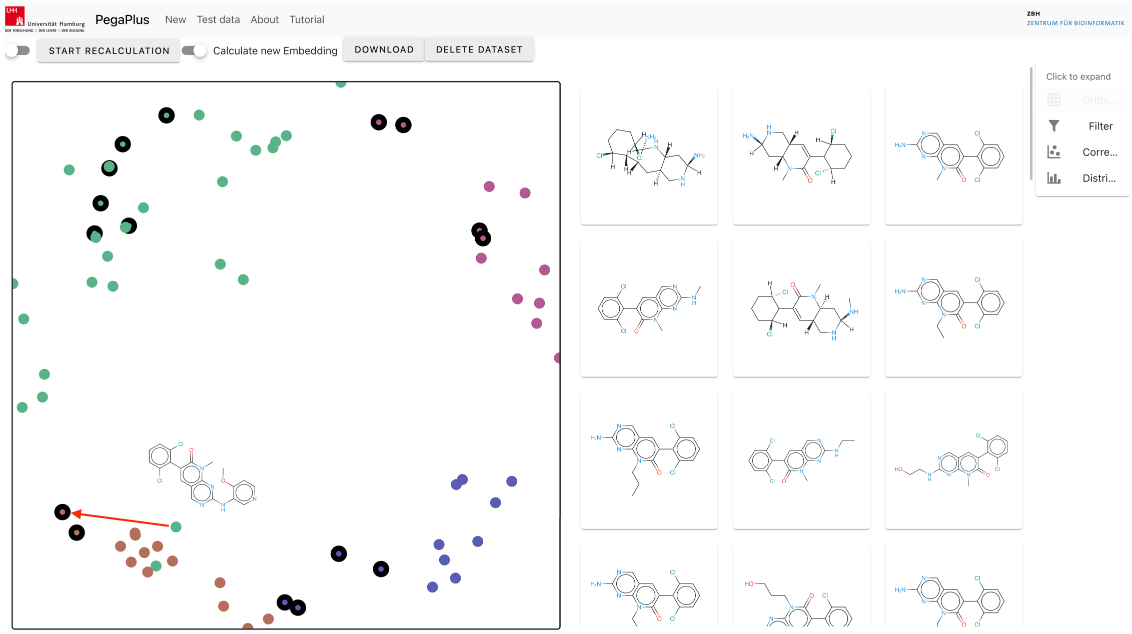

(s) The clustering result is shown after the recalculation. Another molecule has been assigned to the brown cluster.

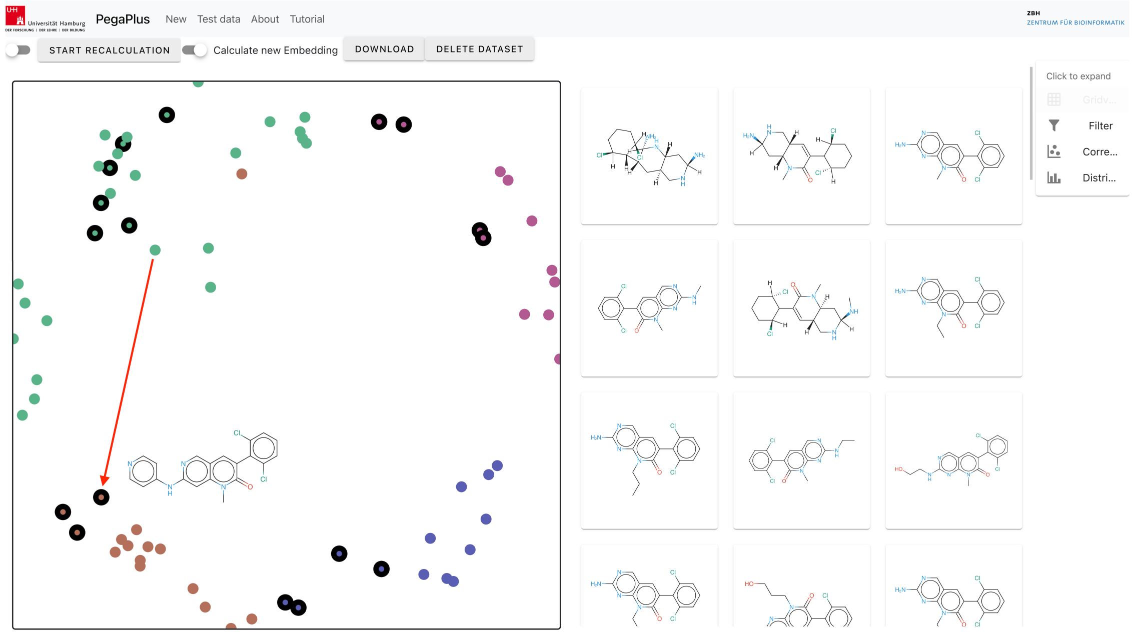

(t) After another calculation, a molecule is reassigned from the green cluster to the brown cluster.

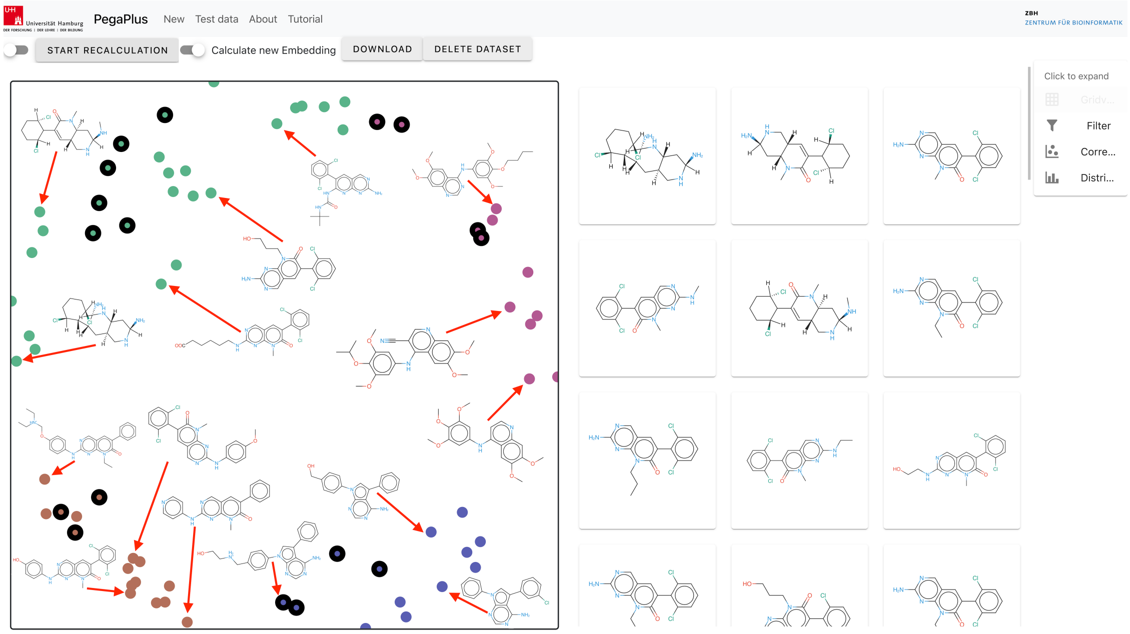

(u) The final clustering result is shown after recalculation.

Figure S11: Figures (a) through (u) illustrate a clustering process aimed at achieving the expert clustering of the SRC dataset. Although the expert clustering contains four clusters, this scenario began with two. Therefore, (a) and (g) also show hidden clusters three and four. In this scenario, seventeen constraints were required. Note that intermediate steps in the clustering process, such as filtering molecules, are not shown. However, this does not mean that these steps were not performed. Molecules are displayed as colored circles on the drawing plane, with their respective cluster colors. Constraints are displayed with thick black borders. The red arrow in (b), for example, indicates motion. In (a), however, it merely indicates the position of the corresponding molecular structure. A circle at the start of an arrow, for instance, in (b) indicates the molecule's initial position, while the circle at the end shows its new position before recalculation.

References

(S1) Willett, P.; Barnard, J. M.; Downs, G. M. Chemical similarity searching. *Journal of chemical information and computer sciences* **1998**, *38*, 983–996.

(S2) Huang, N.; Shoichet, B. K.; Irwin, J. J. Benchmarking sets for molecular docking. *Journal of medicinal chemistry* **2006**, *49*, 6789–6801.

(S3) Good, A. C.; Oprea, T. I. Optimization of CAMD techniques 3. Virtual screening enrichment studies: a help or hindrance in tool selection? *Journal of computer-aided molecular design* **2008**, *22*, 169–178.
